# Structural basis for inactivation of PRC2 by G-quadruplex RNA

**DOI:** 10.1126/science.adh0059

**Published:** 2023-09-21

**Authors:** Jiarui Song, Anne R. Gooding, Wayne O. Hemphill, Brittney D. Love, Anne Robertson, Liqi Yao, Leonard I. Zon, Trista E. North, Vignesh Kasinath, Thomas R. Cech

**Affiliations:** 1 Department of Biochemistry, University of Colorado Boulder, Boulder, CO 80303, USA.; 2 BioFrontiers Institute, University of Colorado Boulder, Boulder, CO 80303, USA.; 3 Howard Hughes Medical Institute, University of Colorado Boulder, Boulder, CO 80303, USA.; 4 Stem Cell and Regenerative Biology Department, Harvard University, Cambridge, MA 02138, USA.; 5 Stem Cell Program, Division of Hematology/Oncology, Boston Children’s Hospital and Dana-Farber Cancer Institute, Boston, MA 02115, USA.; 6 Harvard Medical School, Boston, MA 02115, USA.; 7 Howard Hughes Medical Institute, Harvard Medical School, Boston, MA 02115, USA.

## Abstract

Polycomb repressive complex 2 (PRC2) silences genes through trimethylation of histone H3K27. PRC2 associates with numerous precursor messenger RNAs (pre-mRNAs) and long noncoding RNAs (lncRNAs) with a binding preference for G-quadruplex RNA. In this work, we present a 3.3-Å-resolution cryo–electron microscopy structure of PRC2 bound to a G-quadruplex RNA. Notably, RNA mediates the dimerization of PRC2 by binding both protomers and inducing a protein interface composed of two copies of the catalytic subunit EZH2, thereby blocking nucleosome DNA interaction and histone H3 tail accessibility. Furthermore, an RNA-binding loop of EZH2 facilitates the handoff between RNA and DNA, another activity implicated in PRC2 regulation by RNA. We identified a gain-of-function mutation in this loop that activates PRC2 in zebrafish. Our results reveal mechanisms for RNA-mediated regulation of a chromatin-modifying enzyme.

Many nuclear proteins that bind chromatin also bind RNA molecules (1-3). The binding of RNA has been suggested to facilitate both positive and negative regulation (e.g., recruitment to target sites and enzymatic inhibition, respectively). Polycomb repressive complex 2 (PRC2) is a prominent example of a chromatin modifier known to be regulated by RNA (4, 5). PRC2 is essential for embryonic development and cell differentiation (6, 7). Some tumors are PRC2 dependent (e.g., because of silencing of tumor suppressor genes), making PRC2 a target for cancer therapeutics (8). PRC2 consists of four core protein components: EZH2 (catalytic subunit), EED [binds histone H3 tri-methylated at lysine 27 (H3K27me3)], SUZ12 (provides a platform), and RBAP48 (7). Associating cofactors define two PRC2 subclasses (9, 10), of which PRC2.2, containing AEBP2 and JARID2, is the subject of this study.

PRC2 binds numerous pre-mRNA and long noncoding RNA (lncRNA) transcripts in vitro and in vivo (11-13). This broad RNA recognition can be explained at least in part by PRC2 preferring an RNA G-quadruplex (G4) motif (14-16), which could be ubiquitous in the transcriptome from intramolecular and perhaps even intermolecular assemblies (17). Proposed models of RNA regulation of PRC2 remain disparate. First, in the “handoff” model, PRC2 requires RNA for recruitment and occupancy on a specific subset of targeted chromatin (18, 19). The direct handoff from RNA to DNA is an intrinsic property of PRC2, as shown by recent biophysical analyses (20). Second, the “eviction” model suggests that nascent RNA removes PRC2 from actively transcribed chromatin to restrict nonspecific activity (14, 21-23). Third, in the “inhibitor” model, RNA and nucleosome binding of PRC2 are mutually exclusive, so RNA serves as a direct competitor to prevent PRC2 action (14, 18, 22, 24). Another version of the inhibitor model proposes that RNA exploits a regulatory site on PRC2 to abolish H3K27me3 binding to EED, which consequently eliminates allosteric activation of EZH2 (25). Therefore, structural details of PRC2-RNA interaction have been needed in the field to provide mechanistic insights and coordinate those models.

Cryo–electron microscopy (cryo-EM) and x-ray crystallography have provided visualization of both substrate-free and nucleosome-bound PRC2 complexes (26-34). However, solving a structure of a PRC2-RNA complex has proved challenging. A streptavidin-biotin–affinity EM grid approach has been successfully used in cryo-EM (35), and here we adapt this technique for ribonucleoprotein (RNP) complexes using biotinylated RNA. We found that PRC2 can dimerize following RNA binding with a protein-protein interface composed of EZH2 CXC domains. The structure provides a molecular explanation for how RNA acts as a PRC2 inhibitor, and it suggests a mechanism for RNA facilitation of PRC2 recruitment.

## Structure of G-quadruplex RNA-mediated PRC2 dimer

**Fig. 1.**
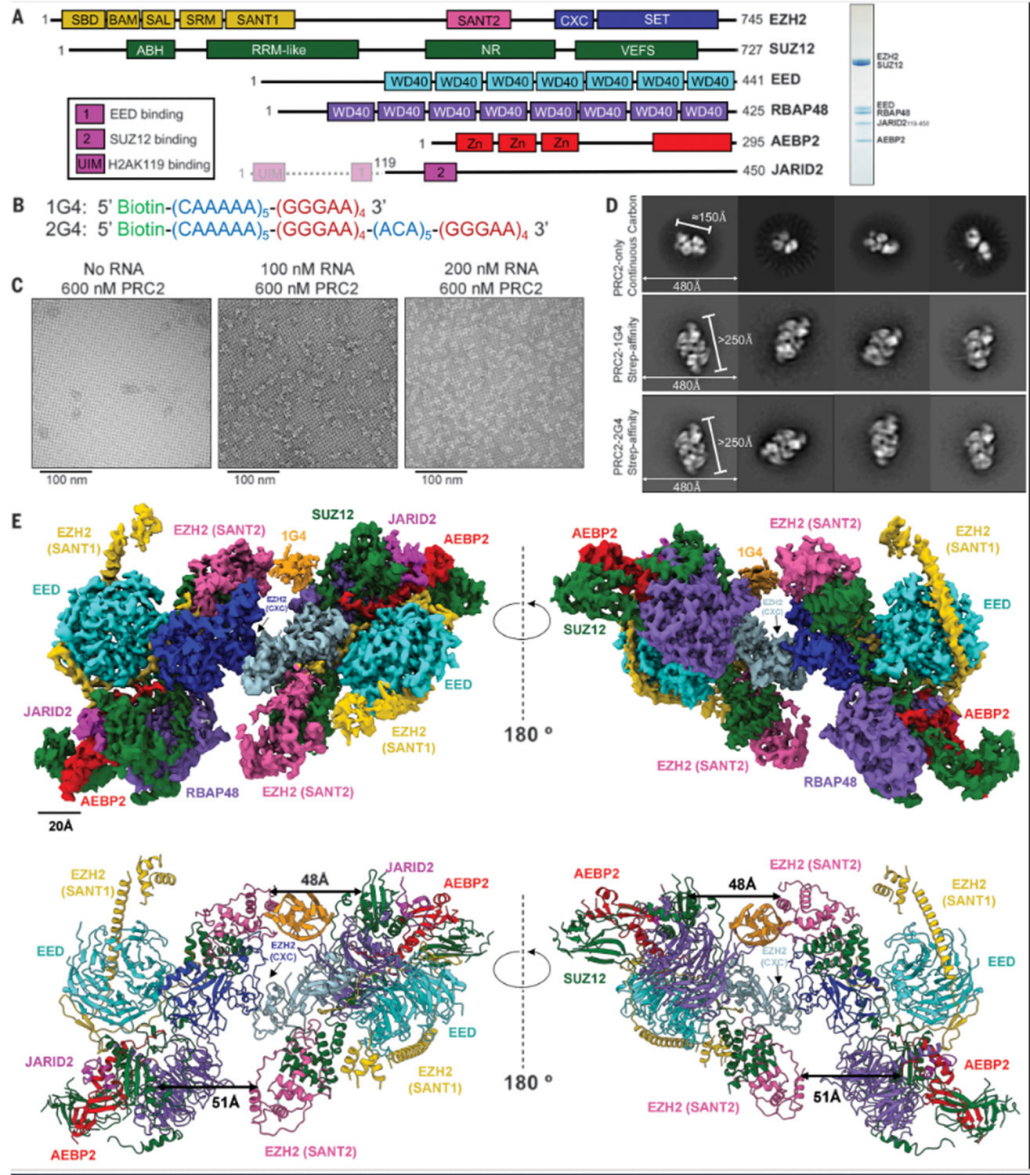
Overall structure of a PRC2-1G4 RNP complex. (**A**) (Left) Schematic of the proteins in the PRC2.2 six-subunit complex. Transparent N-terminal region of JARID2 was not included. (Right) Coomassie-stained gel of purified PRC2. (**B**) The two RNA oligonucleotides used in this study. (**C**) Negative-staining EM images of streptavidin-affinity grids with excess PRC2 and various 1G4 RNA concentrations. (**D**) Negative-staining EM provided 2D-class averages of PRC2 alone collected from continuous carbon grids and PRC2-G4 RNAs from streptavidin-affinity grids. (**E**) (Top) Cryo-EM density map of PRC2 bound to 1G4 RNA. EZH2 (CXC-SET) of protomer 1 in blue, EZH2 (CXC-SET) of protomer 2 in light blue, and 1G4 RNA in orange. (Bottom) The atomic model of PRC2 bound to 1G4 RNA. Distances between EZH2 (SANT2) and SUZ12 (RRM-like) are highlighted by black arrows to emphasize the change from 1G4-binding.

We prepared six-subunit PRC2.2 complexes with full-length EZH2, SUZ12, RBAP48, EED, embryonic short-isoform AEBP2, and truncated JARID2_119–450_ (Fig. 1A), as well as two G4-forming RNAs (Fig. 1B) that bind PRC2 in vitro (fig. S1). We used streptavidin-affinity EM grids to capture 5′-biotinylated RNAs, which select for RNA-bound PRC2 complexes and also protect them from the hydrophobic water-air interface. Because this method had not been applied to an RNP complex, we validated it by testing different RNA concentrations with the same excess of PRC2. The number of particles observed by negative staining was proportional to the RNA concentration in most fields (Fig. 1C), indicating that the vast majority of protein complexes on the grid are bound to RNA. Compared with RNA-free PRC2 (particles ~150 Å in diameter), the majority of two-dimensional (2D) class averages from 1G4- and 2G4-bound PRC2 complexes were larger (particles ~250 Å in diameter), containing two recognizable PRC2 complexes (Fig. 1D).

We determined the cryo-EM structure of the dimeric PRC2-1G4 RNA complex at 3.4-Å resolution from consensus refinement, and at 3.3 Å from multibody refinement (36) (figs. S2 and S3 and table S1). The two PRC2 protomers in the RNP complex are nearly identical and have a conformation previously characterized as the SANT1 extended form (37) (Fig. 1E and fig. S4). We identified a protein interface in the RNA-induced dimer that is a localized EZH2-EZH2 interaction (described in the dimer interface section below) (movie S1).

Notably, this PRC2 dimer has imperfect *C*_2_ symmetry (fig. S5A) associated with differential occupancy of RNA in the two symmetric sites (fig. S5B). The stronger density has a volume representative of a G4 RNA (fig. S5, C and D, and S6D), whereas the symmetric site has discontinuous density and is not discussed hereafter. We could not obtain high-quality RNA density for de novo modeling in either of the sites from multibody refinement (fig. S5, C and D) and particle subtraction classification (fig. S6) (38). We attribute this to the multiple independent and flexible interactions between PRC2 and RNA. The G4 RNA is not nestled into the surface of the protein, as is typically seen for RNA-protein complexes, but instead appears to be separated from the protein. The RNA density is in closest proximity to the EZH2 SANT2 domain (residues 353 to 362), EZH2 CXC domain (residue R532), EZH2 SET domain (residue N697), the RRM (RNA recognition motif)–like domain of SUZ12, and an unstructured region of RBAP48 (residues 92 to 107) (movie S1). These binding sites, excluding RBAP48, are supported by previous in vitro and in vivo studies (25, 26, 39, 40). We were able to observe density that links regions proximal to PRC2 and G4 RNA at 3.9-Å resolution from particle subtraction and classification (described in the arginine-rich site section below).

The distance between the two PRC2 protomers is reduced from 51 to 48 Å on the side with stronger G4 density (Fig. 1E, bottom), which not only explains the imperfect symmetry, but also supports the model of a single G4 being sufficient for PRC2 dimerization. Although the stoichiometry of most RNPs is 2 PRC2:1 RNA in our preparations, we do not reject the possibility of a PRC2 dimer engaging two independent G4 RNAs simultaneously.

## G-quadruplex RNA induces PRC2 dimerization in solution

**Fig. 2.**
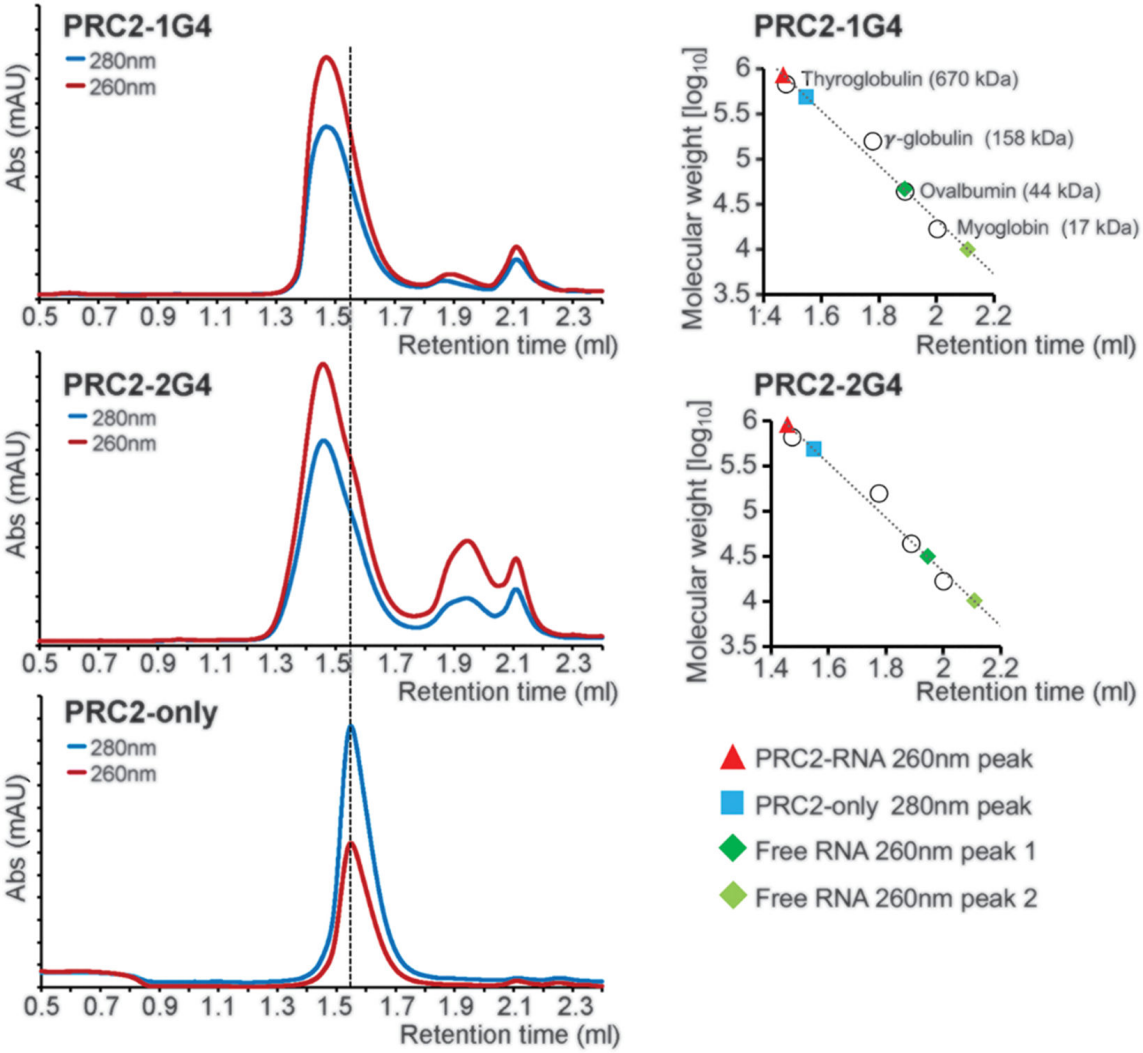
G4 RNA induces PRC2 dimerization in solution. (Left) Size-exclusion chromatography of PRC2 preincubated with 1G4, 2G4, and mock (protein only). Abs, absorbance; mAU, milli–absorbance unit. (Right) Standard curves were used to estimate the molecular weights of PRC2 complexes.

To validate the dependence of PRC2 dimerization on G4 RNA binding in solution, we used analytical size-exclusion chromatography and mass photometry. In the absence of RNA, our six-subunit PRC2 complex chromatographed as a monomer (Fig. 2), which was consistent with an absolute molecular weight of 340 kDa (fig. S7A). Incubating PRC2 with 18-kDa 1G4 RNA or 30-kDa 2G4 RNA led to a large RNP complex of approximately 720 kDa measured by both size-exclusion chromatography (Fig. 2) and mass photometry (fig. S7C), which was consistent with a dimer. In addition, native gel electrophoresis of PRC2-G4 RNP cross-linked with glutaraldehyde indicated that G4 RNA remains bound as part of a cross-linked complex (fig. S7D). Furthermore, negative-staining EM reference-free 2D class averages of cross-linked complexes on conventional carbon-support grids confirmed the presence of RNA-mediated PRC2 dimers as observed in the non–cross-linked streptavidin-affinity grids (fig. S7E). Together, our results verified the requirement of RNA for this specific PRC2 dimerization in solution and provided confidence that the dimer was not an artifact of streptavidin-affinity selection.

To test the specificity of G4 structure in mediating PRC2 dimerization, we used microscale thermophoresis (MST) to measure PRC2-RNA binding in different reaction conditions (fig. S8). In a G4-favoring KCl buffer, the MST profile showed two distinguishable stages of thermo-phoretic mobility. This biphasic binding curve is typical for two binding events (41), which suggests that a higher-affinity binding site on PRC2 is primarily occupied at low PRC2 concentrations (1 PRC2:1 RNA), and at higher PRC2 concentrations, lower-affinity binding of a second PRC2 follows (2 PRC2:1 RNA). In addition, we performed the same MST assays in a G4-destabilizing LiCl buffer or using a G-rich single-stranded RNA with no G4-forming potential. Neither experiment gave a distinct biphasic curve, indicating that PRC2 dimerizes specifically on RNA containing at least one G4 motif.

## The dimer interface prevents nucleosome and H3 tail binding

**Fig. 3.**
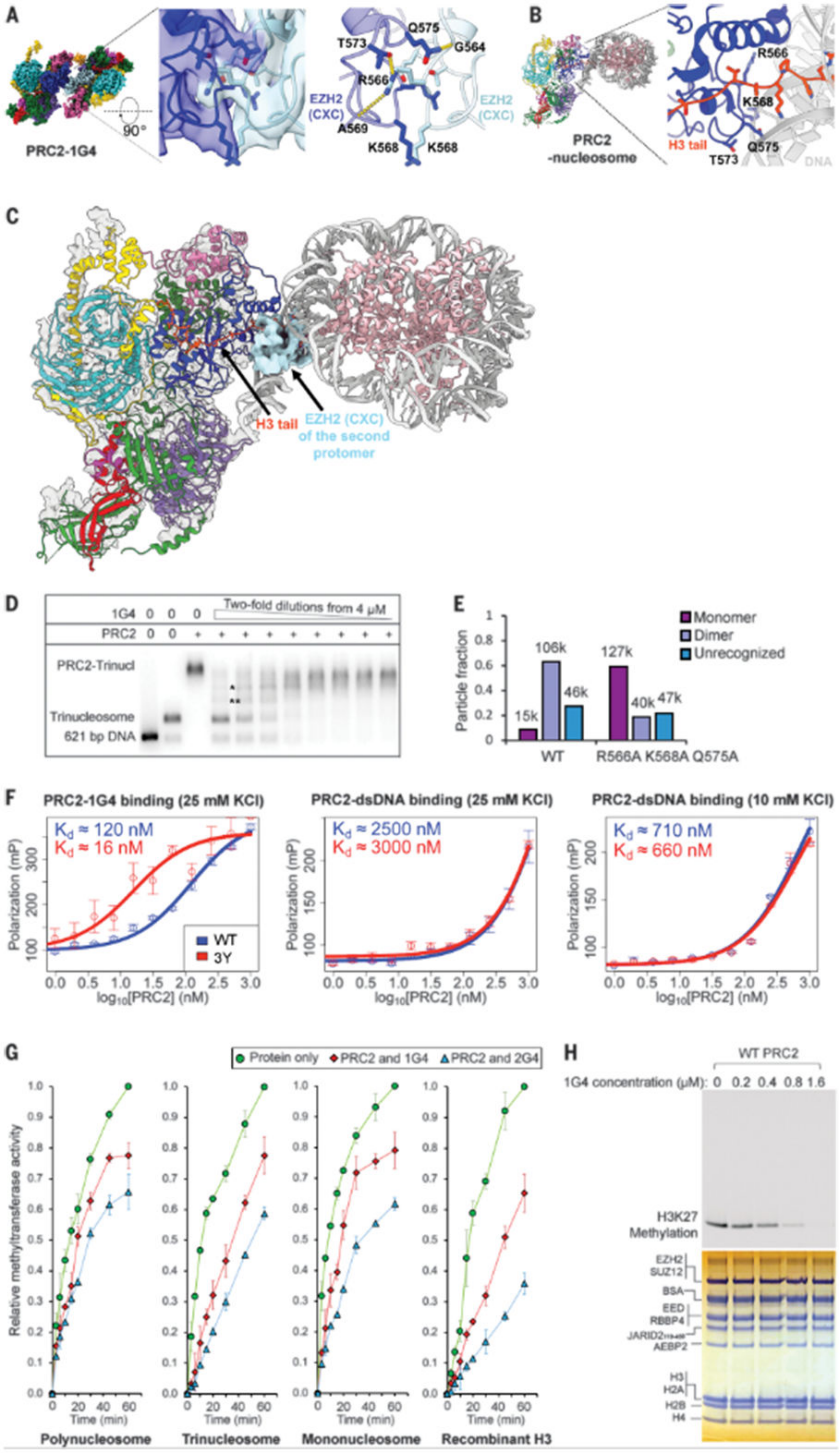
RNA-mediated PRC2 dimer is an inactive complex. (**A**) Cryo-EM structure of PRC2-1G4 complex with zoom-in to show the interface of two EZH2 CXC domains. Interacting residues (R566, K568, T573, and Q575) are highlighted in the stick representation. One set of hydrogen bonds (R566NE-T573OG1, 2.70-Å distance; R566NH1-A569O, 2.73-Å distance; and Q575NE2-G564O, 2.51-Å distance) is indicated by yellow dashed lines. The other set of hydrogen bonds between the same amino acid pairs in the second PRC2 protomer is not shown for clarity. Another view of the same region is shown in fig. S3G. (**B**) Structure of PRC2-nucleosome complex (PDB: 6WKR) with zoom-in to emphasize the CXC interactions with nucleosome H3 tail (orange). The same residues as shown in (A) are highlighted in stick representation. (**C**) Superposition of the EZH2 CXC domain of the RNA-bound PRC2 on the nucleosome-bound PRC2 to emphasize disparate functions of the CXC domain. (**D**) Nucleosome-RNA competition assay. PRC2 was incubated with constant amount of radiolabeled trinucleosome and serial dilutions of 1G4 RNA. Incomplete PRC2-trinucleosome complexes are indicated by * and **. We assume two of three nucleosomes were occupied by PRC2 in *, and one of three nucleosomes in **. (**E**) Negative-stain EM to quantify monomer and dimer particles of EZH2 R566A K568A Q575A binding 1G4. The number of particles in each class is indicated above each bar. (**F**) Binding affinity of 1G4 RNA or dsDNA to WT PRC2 and EZH2 R566Y K568Y W575Y (3Y) measured by FP. We used a reaction buffer with a lower salt concentration to achieve higher binding affinity for the dsDNA (right). K_d_, dissociation constant. (**G**) Methyl-transferase activities from figs. S12 to S15 are plotted against reaction times for PRC2 (400 nM) preincubated with 1G4 (400 nM), 2G4 (400 nM), and mock (protein only). Error bars represent standard deviations of three replicates performed on different days. (**H**) Methylation of trinucleosomes by PRC2 with serial dilutions of 1G4 RNA. (Top) Radiogram that shows methylation. (Bottom) Coomassie-stained gel. Single-letter abbreviations for the amino acid residues are as follows: A, Ala; C, Cys; D, Asp; E, Glu; F, Phe; G, Gly; H, His; I, Ile; K, Lys; L, Leu; M, Met; N, Asn; P, Pro; Q, Gln; R, Arg; S, Ser; T, Thr; V, Val; W, Trp; and Y, Tyr.

The PRC2-1G4 RNA dimer has three features that would be expected to inhibit nucleosome binding and histone methylation. First, the residues within the CXC domain of EZH2 interact with the CXC of the second protomer to form the dimer interface. This dimer interface includes two R566–A569 hydrogen bonds, two R566–T573 hydrogen bonds, two Q575–G564 hydrogen bonds, and a K568–K568 hydrophobic interaction (Fig. 3A). By contrast, in a nucleosome-bound PRC2, the CXC domain facilitates the catalytic activity of the adjacent SET domain of EZH2, specifically with R566, K568, T573, and Q575 contributing to interactions with nucleosome DNA and the H3 tail (Fig. 3B) (27). The disparate functions of the CXC domain in these different PRC2 structures are seen by the superposition of our density map onto the nucleosome-bound PRC2, which shows clashes with both the DNA and the H3 tail (Fig. 3C and movie S2). Therefore, we propose that nucleosome binding and H3 tail loading, both of which are essential for histone methy-transferase (HMTase) activity, are mutually antagonistic with RNA-mediated PRC2 dimerization. The disruption of nucleosome-PRC2 complexes by 1G4 RNA was confirmed by a competition-binding assay in solution (Fig. 3D).

Second, the EZH2 bridge helix (residues 500 to 516)—important for nucleosome DNA binding and channeling H3 tail into the active site of the EZH2 SET domain (27)—is disordered in both protomers of our RNP complex (fig. S9). This is consistent with structures of PRC2 lacking nucleosome substrate. Lastly, the EZH2 C-terminal helix (residues 738 to 742), which points away from the H3K27 binding site in nucleosome-bound PRC2, now occludes the active site in RNA-bound PRC2 (fig. S9) and appears to serve as an additional mechanism to prevent H3 tail binding.

To test the importance of the CXC dimer interface, we purified a mutant (EZH2 R566A K568A Q575A). Mutation of these residues did not affect G4 RNA binding (fig. S10, A and B) or prevent RNA-induced dimerization (fig. S10, C and D). However, negative-staining EM with streptavidin-affinity grids classified substantially more monomer-size particles from the mutant (59%) than the wild-type (WT) PRC2 (9%) (Fig. 3E and fig. S10E). This suggests that these EZH2 mutations impacted the overall stability of the PRC2 dimer by destabilizing the protein-protein interaction, and consequently, one protomer more easily dissociated during stringent washes (dilutions) in our grid preparations compared with the WT. Because streptavidin-affinity grids only retain RNA-bound complexes, those monomer particles of the mutant could also represent an intermediate stage of one PRC2 engaging RNA prior to association of the second PRC2, which is consistent with the biphasic binding curve observed in our MST assays.

We next attempted to disrupt the CXC interface more severely by substituting bulky sidechains of tyrosine, so we constructed an EZH2 R566Y K568Y Q575Y mutant. Unexpectedly, this mutant had higher binding affinity to the G4 RNAs, as determined by fluorescence polarization (FP) assays (Fig. 3F, left) and electrophoretic mobility-shift assays (EMSA) (fig. S11, A and B). Notably, double-stranded DNA (dsDNA) binding of this mutant was not affected (Fig. 3F, middle and right), which further indicates that DNA and RNA use separate mechanisms to engage PRC2 even though they bind mutually antagonistically. Although this mutant PRC2 is a monomer as observed by negative-staining EM, RNA-bound particles showed a dominant population of dimer complexes, which is consistent with the increased RNA binding affinity and the role of RNA in mediating PRC2 dimerization (fig. S11, C and D). We propose that the aromatic sidechains of tyrosine might stack on each other and therefore stabilize the CXC interface. Thus, the dimerization interface need not be specific, and it appears to be RNA binding rather than protein-protein interaction that drives PRC2 dimerization. Overall, we observed a positive correlation between the CXC dimer interface and G4 RNA binding.

## RNA-induced PRC2 dimer is inactive

Structural observations on the EZH2 CXC interface prompted us to hypothesize that the HMTase activity of the G4-induced PRC2 dimer would be inhibited. To test this, we performed activity assays to compare free PRC2 with RNA-sequestered dimers (Fig. 3G and figs. S12 to S15). As expected, we detected substantial reductions in methylation rates with all substrates (including recombinant H3) in response to RNA binding, with stronger inhibition by the higher-affinity 2G4 RNA (Fig. 3G). The extent of inhibition was limited by the RNA concentration because complete inhibition was achieved with excess 1G4 RNA (Fig. 3H and fig. S16A).

## An arginine-rich site of EZH2 binds G4 RNA and participates in RNA-to-DNA handoff

**Fig. 4.**
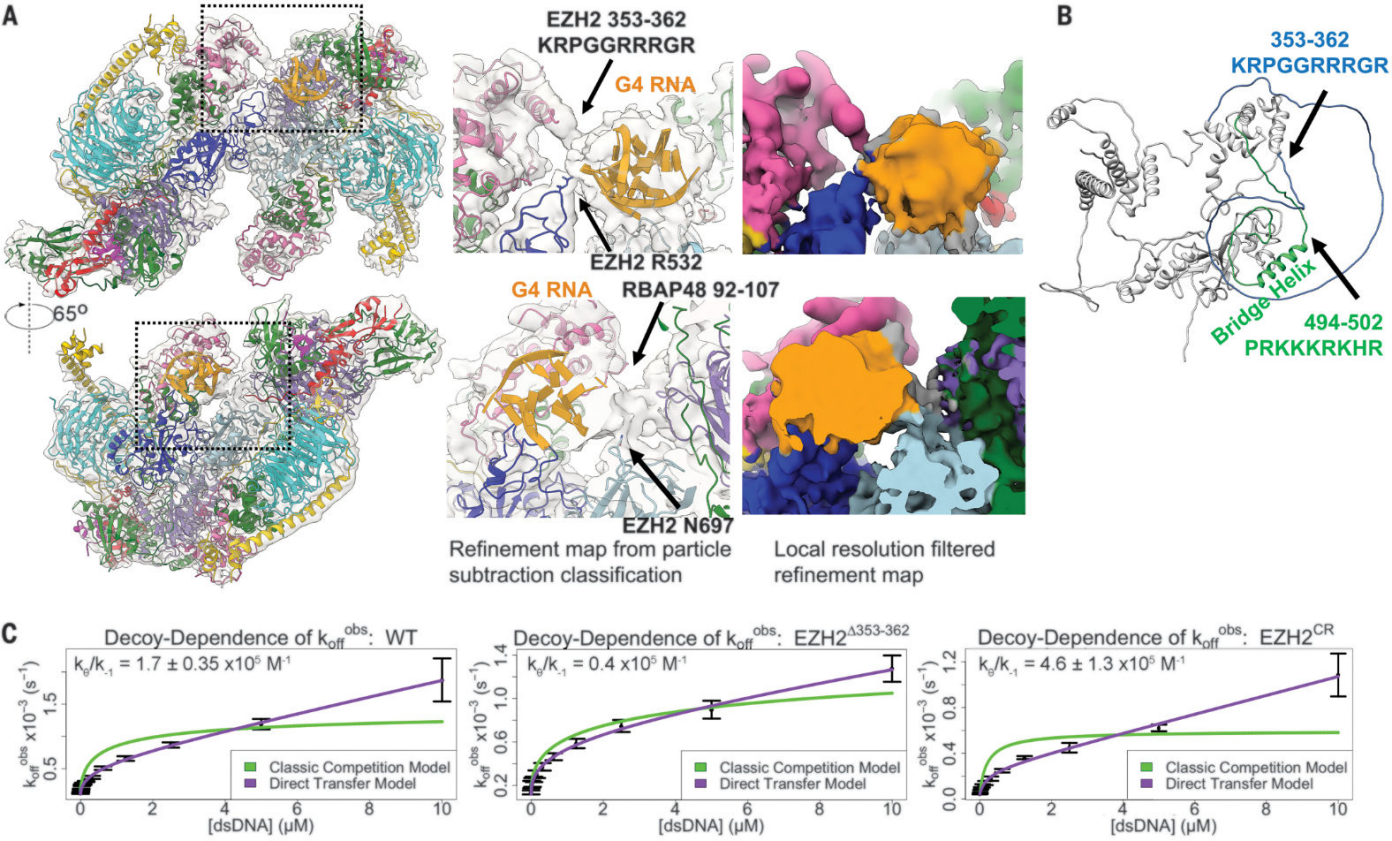
EZH2 loops physically contact G4 RNA and contribute to direct handoff from RNA to DNA. (**A**) Map of PRC2-1G4 RNA from particle subtraction and classification (fig. S6) with zoom-ins to emphasize the observed physical interactions of PRC2 and G4 RNA. (**B**) EZH2 structure from AlphaFold predicts two disordered loops of EZH2. Arginine-rich loop [EZH2(353–362)] and lysine-rich loop [EZH2(494–502)] are indicated in blue and green, respectively. (**C**) FP assays to monitor the transfer kinetics of PRC2 from fluorescently labeled 1G4 RNA to a dsDNA competitor. The ratio k_θ_/k_−1_ of the PRC2 RNA-to-dsDNA direct-transfer rate constant (k_θ_) and the PRC2-RNA dissociation rate constant (k_−1_) provides a measure of the propensity of PRC2 to exchange these ligands by the direct transfer mechanism. WT PRC2 had k_θ_ = 90 ± 11 M^−1^s^−1^, k_−1_ = 5.6 ± 0.49 × 10^4^ s^−1^, and k_θ_/k_−1_ = 1.7 ± 0.35 × 10^5^ M^−1^. EZH2 Δ353–362 had k_θ_ = 48 M^−1^s^−1^, k_−1_ = 12 × 10^4^ s^−1^, and k_θ_/k_−1_ = 0.4 × 10^5^ M^−1^. EZH2 CR had k_θ_ = 130 ± 62 M^−1^s^−1^, k_−1_ = 2.7 ± 0.63 × 10^4^ s^−1^, and k_θ_/k_−1_ = 4.6 ± 1.3 × 10^5^ M^−1^. k_off_^obs^, dissociation rate constant observed.

Applying particle subtraction and classification (fig. S6), we identified multiple sites in EZH2 [EZH2(353–362): KRPGGRRRGR, EZH2 R532, and EZH2 N697] that physically contact G4 RNA (Fig. 4A). The arginine-rich EZH2(353–362) has been implicated in binding lncRNA (25, 39), and similar arginine-rich sequences in multiple transcription factors have been linked to RNA binding (42). EZH2 truncation [EZH2(Δ353–362)] and a local charge-reversed EZH2 [EZH2 CR(353–362): DEPGGEEEGE] both exhibited decreased HMTase activity but showed no obvious reduction in G4 RNA binding or G4 RNA-mediated PRC2 dimerization in vitro (fig. S17). We also generated a double-truncation mutant [EZH2(Δ353–362 Δ494–502)] to remove an adjacent lysine-rich site (EZH2 494–502) (Fig. 4B). EZH2 residues 494 to 502 were previously implicated in binding nucleosome DNA and G4 RNA (27, 40) and might compensate for RNA binding in the absence of EZH2 residues 353 to 362. PRC2 containing EZH2(Δ353–362 Δ494–502) exhibited a 1.5- to twofold reduction of binding affinity for G4 RNA (fig. S17G). We attribute this modest reduction to the presence of other RNA-binding regions observed in our structure and supported by previous studies (25, 40, 43).

PRC2 has the intrinsic ability to directly transfer or hand off from RNA to DNA, without there ever being a free-enzyme intermediate (20, 44). We therefore examined whether the arginine-rich EZH2(353–362) region, in addition to its role in RNA binding, is important for such RNA-to-DNA handoff. We found that the EZH2(Δ353–362) and EZH2 CR mutants had a 4.3-fold reduction and a 2.7-fold increase, respectively, in the propensity for direct transfer from RNA to DNA (Fig. 4C). We rationalized these results with a model (fig. S18) in which PRC2 harbors the arginine-rich EZH2(353–362) and lysine-rich EHZ2(494–502) to form a ternary intermediate with both RNA and DNA binding. EZH2(Δ353–362) and CR mutations of the RNA-binding region affect the propensity for direct transfer in different directions.

## EZH2 CR is a gain-of-function mutant in zebrafish development

**Fig. 5.**
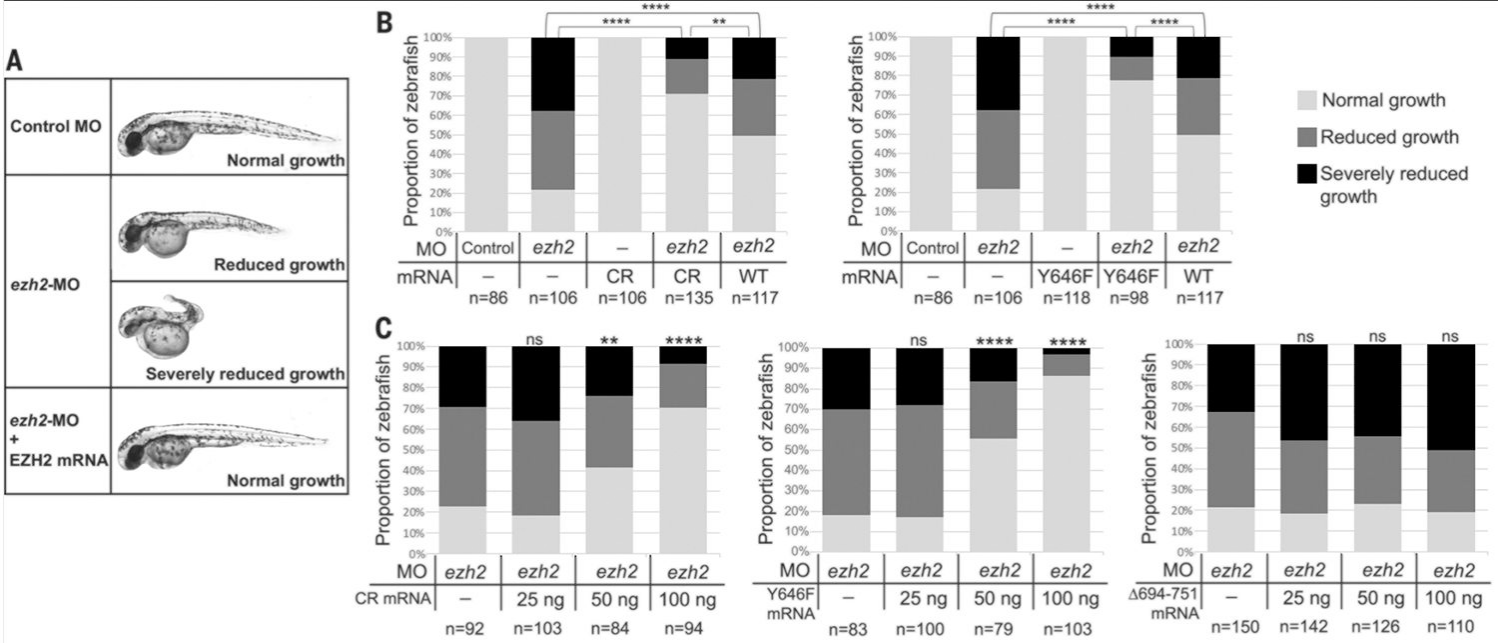
EZH2 CR is a gain-of-function mutant in rescue of zebrafish development. (**A**) Representative images of injected zebrafish embryos at 48 hours post fertilization (hpf). Gross phenotypic scoring of anterior-posterior axis growth was sorted into three categories: normal, reduced, and severely reduced growth. (**B**) Scoring of anterior-posterior axis growth at 48 hpf. Zebrafish embryos were injected with 4-ng *ezh2*-MO or the same amount of control MO. For rescue experiments, 100 ng of mRNA encoding WT or mutated human EZH2 was coinjected with *ezh2*-MO. At least three clutches were examined for each injection. Fisher’s exact test was used to determine the *P* values. (**C**) Dose-response experiments with 25, 50, and 100 ng of mRNA coinjected. Statistical analyses were performed as in (B) by comparing different doses with *ezh2*-MO alone. ns, not significant; *P < 0.05; **P < 0.01; ***P < 0.001; ****P < 0.0001.

We used zebrafish to examine the significance of the EZH2 G4 RNA-binding sites in vertebrate development. Zebrafish and human EZH2 proteins have high sequence identity, including the regions responsible for G4 RNA binding and RNA-induced PRC2 dimerization (fig. S19A). EZH2 knockdown in zebrafish by antisense morpholino oligonucleotides (MOs) led to a severe growth defect (45) represented by gross alterations in the length of the anterior-posterior axis (Fig. 5A). As expected, coinjection with mRNA that encodes human WT EZH2 significantly rescued the growth defects (*P* < 0.0001) (Fig. 5B and fig. S19B). Mutant EZH2 mRNAs rescued overall development to varying degrees (fig. S19C). Notably, the EZH2 CR mutant had a gain-of-function phenotype, giving significantly better rescue than that of WT EZH2 (P < 0.01) and phenotypically mimicking a gain-of-function mutant (EZH2 Y646F) that is well-studied in the human system and frequently found in lymphoma (Fig. 5B) (46-48). Dose-response assays by coinjecting *ezh2*-MO and increasing amounts of mRNAs (Fig. 5C) confirmed that the extent of rescue was consistently similar between CR and Y646F, whereas the catalytically dead EZH2(Δ694–751) gave no rescue. Therefore, the EZH2 CR mutant, which shows an increased propensity for direct transfer between RNA and DNA in vitro, also behaves as a gain-of-function mutant in zebrafish development.

## Discussion

In the past 10 years, lncRNAs and pre-mRNAs have become prominent in discussions of PRC2 regulation (4, 5). Because of the broad PRC2 transcriptome (11, 12), deciphering molecular details of PRC2-RNA interaction has been challenging. PRC2 binds G4 RNA in vitro (14, 15, 23), PRC2 binding sites on chromatin genome wide are associated with G-tract motifs (15), and a well-defined G4-forming RNA TERRA (telomeric repeat–containing RNA) recruits PRC2 to telomeres (16). Thus, we focused on G4 RNA in the present study.

By using a biotinylated G4 RNA with streptavidin-biotin affinity grids, we determined the cryo-EM structure of an RNA-bound PRC2 complex. This structure supports the earlier conclusion that nucleosomal DNA and RNA binding are mutually antagonistic (22, 24), but it provides a much more interesting mechanism than just competition on overlapping sites. Instead, G4 RNA triggers formation of a PRC2 dimer that occludes the DNA-binding amino acids. Based on the present structure and biochemical and biophysical data, we propose a model that can explain multifaceted functions of RNA in PRC2 regulation.

First, actively transcribed loci, which need to avoid silencing by PRC2, generate nascent RNA transcripts that have the potential to dimerize PRC2 among other RNA processing events. The RNA-induced dimer simultaneously inactivates two PRC2 complexes (no H3 tail binding) and evicts them from local chromatin (no nucleosome DNA binding). Residues of the EZH2 CXC domain, known to load the H3 tail into the catalytic groove, are occupied in a protein-protein interaction that reinforces the dimerization. Dimerization may prevent the spreading of H3K27me3 across regions near preexisting H3K27me3 or JARID2 116me3 marks, both of which induce allosteric activation of PRC2 (31, 49, 50), and therefore, it may help define heterochromatin boundaries.

Second, the interactions of PRC2 with RNA are proposed to be important for PRC2 occupancy on chromatin (19, 51). Consistent with this idea, PRC2 has the intrinsic ability to translocate onto target chromatin as it simultaneously dissociates from inhibitory RNA (20). To reconcile the role of RNA in inhibiting and evicting PRC2 from chromatin and its role in promoting PRC2 chromatin occupancy, we propose that EZH2 harbors partially redundant nucleic acid binding sites that allow PRC2 to transiently engage both RNA and DNA, thereby facilitating direct transfer from RNA to DNA. The EZH2 CR mutant, designed to destabilize RNA binding, enhances the direct transfer of PRC2 from RNA to DNA in vitro. This CR mutant rescues the knockdown of PRC2 in zebrafish better than WT EZH2. This gain-of-function phenotype is consistent with a direct transfer from RNA to DNA, which facilitates PRC2 activity in vivo. However, the many differences between in vitro and in vivo experiments warrant a cautious interpretation. The precise mechanism of the EZH2 CR mutant gain-of-function merits further investigation.

Our structure describes one mode of RNA recognition by PRC2, but there may very well be others. Other reported RNA-binding sites include the RNA-binding region (RBR) adjacent to the bridge helix of EZH2 (residues 494 to 502) (40), the stimulatory recognition motif (SRM) of EZH2 (residues 127 to 153) (25), the EED amino acids close to EZH2 SRM (residues 336 to 355) (25), and the JARID2 RBR (residues 332 to 358) (43). Although we did not obtain any subclass map having a distinguishable RNA density in proximity to those regions, this is not sufficient to reject their RNA-binding potential. We propose that the numerous RNA-binding regions within PRC2 explain why mutations give only modest effects on RNA binding in this and other studies.

PRC2 has been shown to dimerize without RNA binding. We consistently find that, in the absence of RNA, a small fraction of PRC2 molecules self-associate into dimers at high protein concentrations, as found for a four-subunit PRC2 holoenzyme (52) and the six-subunit PRC2.2 complex (fig. S20A). However, 2D class averages of self-associated dimers show a different dimer interface than the RNA-induced dimer (fig. S20B). In addition, two reported domain-swapped PRC2 dimers—PRC2-PCL and PRC2:EZH1 (29, 32)—have completely different architectures than our RNA-mediated dimer.

Aside from PRC2, many chromatin-associated complexes have been found to interact with RNA, including other histone modifiers (2), transcription factors (42, 53, 54), and DNA methyltransferase (55, 56). Similar to PRC2, they tend not to have canonical RNA-recognition motifs and bind RNA broadly, and obtaining molecular structures of the RNA-protein complexes has been very challenging. Ultimately, solving additional structures of RNA bound to epigenetic modifiers will reveal the mechanisms by which RNA serves direct regulatory roles, rather than simply serving as a messenger.

## Supplementary Material

Movie 1

Movie 2

Supplementary

Reproducibility Checklist
