# Supplementary material for: Structural basis for inactivation of PRC2 by G-quadruplex RNA
Source: Science. Author manuscript; Available in PMC 2024 Jun 21. (PMC11191771; doi:10.1126/science.adh0059)
Supplement: Supplementary [file NIHMS1998120-supplement-Supplementary.pdf]

Science
AAAS

Supplementary Materials for

Structural basis for inactivation of PRC2 by G-quadruplex RNA

Jiarui Song *et al*.

Corresponding authors: Vignesh Kasinath, vignesh@colorado.edu; Thomas R. Cech, thomas.cech@colorado.edu

Science **381**, 1331 (2023)
DOI: 10.1126/science.adh0059

The PDF file includes:

Materials and Methods
Figs. S1 to S20
Table S1
References

Other Supplementary Material for this manuscript includes the following:

Movies S1 and S2
MDAR Reproducibility Checklist

Materials and Methods

Protein expression and purification

Full-length EED, SUZ12, RBAP48, His-tagged EZH2 isoform 2 (UniProt Q15910-2), Strep-GFP-tagged embryonic isoform of AEBP2, and Strep-GFP-tagged truncated JARID2 (amino acids 119-450) were assembled into a single multi-bac plasmid. Each expression cassette has independent promoter and terminator. This multi-bac plasmid was used to make infectious baculovirus stock in Sf9 (*Spodoptera frugiperda*, IPLB-Sf-21-AE) cells using the Bac-to-Bac system (Invitrogen). To express recombinant complex, HighFive (*Trichoplusia ni*, BTI-Tn-5B1-4) cells were transfected with baculovirus at 28°C for 66 hours, washed with cold PBS buffer, and frozen in liquid nitrogen until use.

All purification steps were performed in a 4°C cold room. Cells were lysed in lysis buffer (25 mM HEPES pH 7.9 at 4°C, 250 mM NaCl, 2 mM $MgCl_2$, 1 mM TCEP, 10 mM imidazole, 0.5% NP-40, 10% glycerol, and protease inhibitor cocktail) for 1 hour and sonicated with mild strength. Debris was then removed by centrifugation at 15,000 rpm for 35 minutes. The supernatant was incubated with Ni-NTA agarose resin (Qiagen) for 1 hour, and resin was washed with 10 column volumes (CV) of lysis buffer, 10 CV of high-salt wash buffer (25 mM HEPES pH 7.9 at 4°C, 1 M NaCl, 2 mM $MgCl_2$, 1 mM TCEP, 0.01% NP-40, and 10% glycerol), and 20 CV of low-salt wash buffer (25 mM HEPES pH 7.9 at 4°C, 150 mM NaCl, 2 mM $MgCl_2$, 1 mM TCEP, 30 mM imidazole, and 10% glycerol). Proteins were then eluted in elution buffer (25 mM HEPES pH 7.9 at 4°C, 150 mM NaCl, 2 mM $MgCl_2$, 1 mM TCEP, 300 mM imidazole, and 10% glycerol) and dialyzed for 2 hours in buffer (25 mM HEPES pH 7.9 at 4°C, 150 mM NaCl, 2 mM $MgCl_2$, 1 mM TCEP, and 10% glycerol) to remove imidazole. After concentrating to 3-5 mg/ml, proteins were incubated with TEV protease overnight. We used an AKTA-FPLC system for subsequent purification with a HiTrap Heparin HP column (Cytiva) and a Superose 6 increase 10/300 column (GE Healthcare). Heparin column was equilibrated with buffer I (20mM HEPES pH 7.9 at 4°C, 150 mM NaCl, 2 mM $MgCl_2$, 1 mM TCEP, and 10% glycerol), and sample was eluted with a linear gradient of buffer II (20mM HEPES pH 7.9 at 4°C, 2 M NaCl, 2 mM $MgCl_2$, 1 mM TCEP, and 10% glycerol). The Superose 6 increase 10/300 column was equilibrated and performed with final storage buffer (25 mM HEPES pH 7.9 at 4°C, 150 mM KCl, 2 mM $MgCl_2$, 10% glycerol, and 1mM TCEP). Protein complex was flash frozen in liquid nitrogen as single-use aliquots and stored at −80°C.

For preparations of mutated PRC2 complexes, Q5 site-directed mutagenesis (NEB) and plasmid synthesis (GenScript) were used to generate constructs containing a single EZH2 expression cassette with corresponding mutations. Then NEBuilder HiFi DNA assembly (NEB) was applied to assemble the final multi-bac plasmids with mutated EZH2 and other PRC2 subunits.

RNP complex assembly

We purchased two G-quadruplex (G4)-forming RNA oligos, 1G4 and 2G4, from IDT RNA oligonucleotide synthesis including HPLC purification service. 1G4 is a 50-nt single-G4 RNA with a 5’ biotin modification, followed by a 30-nt A-rich linker sequence to provide flexibility between biotin and the functional G4 group. $(GGGAA)_4$ sequence folds into a stable G4 structure. 2G4 has two-independent-G4 motifs separated by a 15-nt A-rich linker, 85 nt total. A-rich sequences were chosen for the linker because poly(A) RNA does not interact with PRC2

(*15*). G4 RNA was heated at 95°C for 3 min, snap-cooled on ice for 5 min, refolded in RNP complex buffer (25 mM HEPES pH 7.9 at 4°C, 50 mM KCl, 2 mM $MgCl_2$, 10% glycerol, and 1mM TCEP) at 37°C for 30 min. PRC2 and S-adenosyl homocysteine (SAH) were added into the reaction at final concentration of 600 nM and 40 µM correspondingly, and the reaction was incubated at 30°C for 30 min to assemble the RNP complex.

Cryo-EM sample preparation

Quantifoil Au 2/2 streptavidin-affinity grids were made in-house using procedures previously described (*35*). Grids were re-hydrated by EM preparation buffer I (25 mM HEPES pH 7.9 at 4°C, 50 mM KCl, 2.5% glycerol, and 1mM TCEP) at room temperature (RT) for 1 hour. After removing the remaining buffer, 4 µl of the assembled RNP complex was applied to the streptavidin-affinity grid. 1G4 RNA was chosen for cryo-EM sample preparation because it exhibited the same 2D projections as 2G4 and avoided the potential complications in sample heterogeneity due to multiple PRC2 binding to a single 2G4 RNA containing two G-quadruplex motifs. The grid was incubated for 5-10 min in a humidified chamber, then washed with 40 µl of EM preparation buffer I and EM preparation buffer II (25 mM HEPES pH 7.9 at 4°C, 50 mM KCl, 2.5% glycerol, 0.01%NP-40, and 1mM TCEP). After the washes, the buffer was wicked away using Whatman filter paper, and 4 µl of the EM preparation buffer II was added immediately. The grid was then transferred to the Leica EM GP2 plunge freezer and blotted for 2-3 s at 8°C and 90% humidity and then plunged into liquid ethane.

Negative staining of the streptavidin-affinity grid was applied through the same protocol. Instead of using a plunge freezer, 5 droplets of 40 µl stain were used. For negative staining using continuous carbon grids, 4 µl of 100 nM protein or 100 nM crosslinked (0.1% glutaraldehyde for 25 min) complex was applied to glow-discharged carbon film 400 mesh Cu grid and incubated for 20 s before staining.

Cryo-EM data collection and processing

Cryo-EM dataset was collected on a Titan Krios equipped with Gatan K3 direct detector in super-resolution mode and a Cs-corrector. A GIF quantum energy filter was used for collection with a 20-eV slit width. Movies were recorded at a nominal magnification of 81,000x, corresponding to a calibrated pixel size of 0.844Å (super-resolution 0.422 Å). Data acquisition was performed using SerialEM for automated data collection with a defocus range of −2.0 to −0.6 µm. The total dose for our dataset was 60 electrons per square angstrom ($e^-/Å^2$). It was acquired as dark-subtracted, non–gain corrected movies, and gain correction was applied during motion correction using MotionCor2 (*57*).

Negative staining datasets were collected on a Tecnai F20 microscope operated at 200 kV, with a Gatan K3 direct detector, at a nominal magnification of 25,000x, corresponding to 1.449 Å per pixel. Most datasets were collected using a dose of 60 $e^-/Å^2$ on continuous carbon grids and streptavidin-affinity grids with >2.5 nm carbon supports. Dataset presented in Fig. S10 was collected at 20 $e^-/Å^2$, because this sample was applied to a streptavidin-affinity grid with an approximately 1 nm carbon support that could not tolerate 60 $e^-/Å^2$.

Data were processed in RELION 4 (*58*). The movie frames were aligned using MotionCor2 (*57*) and CTF parameters were fit using CTFFIND (*59*). The background streptavidin lattice of each

micrograph was subtracted using in-house scripts (*35*). LoG automatic picking was applied to pick individual particles. Initial models were generated within RELION from negative staining data and used as reference for the first round of 3D classification. Subsequent processing steps including several runs of regular 3D classification and 3D classification without alignment (regularization parameter T=24), which used references from previous good classes. The selected 217,196 particles were then re-extracted and subjected to per-particle defocus refinement, beam-tilt refinement, 3D refinement, and postprocessing to generate the consensus map. Soft-edged masks used in multibody refinement (*36, 60*) were generated within RELION. Particle subtraction and classification was applied as described in (*38*). Local resolution estimation was performed using the same soft, spherical masks used during refinement.

<u>Model building</u>

Individual PRC2 protomers were built using cryo-EM maps from the multibody refinement. The coordinates of nucleosome-bound PRC2 six-subunit complex (PDB: 6WKR) (*27*) was used as a starting model from which all the coordinates were adjusted and rebuilt in the new map using COOT (*61*). We used EZH2 isoform 2 (UniProt Q15910-2, 297-298:HP→HRKCNYS) in this study, which has a five-residue insertion that forms an unstructured loop, instead of the more frequently used isoform 1. This insertion only extended the length of loop 297-303 without altering other defined secondary and tertiary structures of EZH2 (fig. S4C). To allow comparison with published PRC2 structures, EZH2 residue numbers in this study are corresponding to isoform 2. The region corresponding to EZH2 isoform 2 was built de novo into the EM density in COOT. 1G4 model was adapted from PDB: 2M18 and then docked into our map for the position of strong RNA density in the multibody refinement map and particle subtraction classification map. We ignored the very weak RNA density. The model of each individual PRC2 promoter was subjected to global refinement and minimization in real space using PHENIX (*62*). These were then subjected to manual inspection and adjustment in COOT followed by refinement again in PHENIX. Combining two protomers and RNA, the model of the final RNP was then refined in PHENIX against the full consensus map with local grid search to validate the interface rotamers (*63*). The cryo-EM density maps and the molecular graphics were prepared with Chimera and ChimeraX (*64*). The distance between PRC2 protomers was calculated using residues from EZH2 SANT2 domain and SUZ12 RRM-like domain. The EZH2 model in Fig. 4B was from the AlphaFold prediction (*65*).

<u>Electrophoretic mobility shift assay (EMSA)</u>

EMSA was conducted as previously described (*40*) with modifications. 1G4 and 2G4 RNA oligos were purchased from IDT without 5’ biotin modification. After 5’ end-labeling with gamma-^{32}P-ATP, oligos were heated, snap-cooled, and refolded in EMSA binding buffer (50 mM Tris-HCl pH 7.5 at 25°C, 100 mM KCl, 2.5 mM $MgCl_2$, 0.1 mM $ZnCl_2$, 2 mM 2-mercaptoethanol, 0.1 mg/ml BSA, 0.1 mg/ml fragmented yeast tRNA, and 5% glycerol). Protein samples were diluted and incubated with refolded RNA at 30°C for 30 min. Samples were loaded to 1% agarose gel (SeaKem GTG Agarose) buffered with 1X TBE and resolved at 66 V for 90 min in a 4°C cold room. Gels were vacuum dried for 60 min at 80°C. Dried gels were exposed to phosphorimaging plates and signal acquisition was performed using a Typhoon Trio phosphorimager (GE Healthcare). Signal intensities were quantified by ImageQuant TL. Data were plotted to calculate Kd values using the Prism software.

Analytic size-exclusion chromatography

In a 50 µl reaction, PRC2 and refolded G4 RNA were mixed at a final concentration of 2 µM with RNP complex buffer (25 mM HEPES pH 7.9 at 4°C, 50 mM KCl, 2 mM $MgCl_2$, 10% glycerol, and 1mM TCEP). The reaction was incubated at 30°C for 30 min to complete RNP assembly and then injected into a Superose 6 increase 3.2/300 column (Cytiva) pre-equilibrated with the RNP complex buffer. The column was run at a flow rate of 0.02 ml/min, monitored by UV260 and UV280 detectors. Gel filtration standard (Bio-rad) was injected and run using the same protocol to estimate the molecular weight of unbound PRC2 and G4-bound RNP complexes.

Mass photometry

1 µM PRC2 and RNA of various concentrations were incubated in MP buffer (25 mM HEPES pH 7.9 at 4°C, 50 mM KCl, 2 mM $MgCl_2$, 5% glycerol, and 1mM TCEP) at 30°C for 30 min to achieve complex assembly. Glutaraldehyde was added at final concentration of 0.1% and reactions were incubated on ice for 25 min prior to quenching with 25 mM Tris (pH 7.5 at RT). Samples were diluted to 500 nM of PRC2 in MP buffer temporarily.

Mass photometry measurements were performed using a Tow MP mass photometer (Refeyn LTD, Oxford, UK). Data were acquired using the Acquire MP software package and analyzed using the Discover MP software, both from Refeyn. MP buffer was prepared fresh on the day of the experiment and checked by Mass Photometry for cleanliness. Glass coverslips (24 × 50 mm, Thorlabs Inc.) were washed with fresh MiliQ-water and then HPLC grade isopropanol and dried with a clean stream of compressed air. Sample chambers were assembled by placing clean 6-well silicon gaskets (Refeyn LTD, Oxford, UK) on the cleaned coverslips. Final samples of cross-linked complexes were prepared directly in one well. The coverslip with gasket was positioned on the stage of the mass photometer. 15 µl MP buffer were added, and the mass photometer was focused. 1.5 µl of 500 nM sample were applied to the well and gently mixed. Therefore, the final PRC2 concentration was 45 nM during measurements. Data acquisition was started immediately to record a 60 s movie. The mass calibration was achieved using beta-amylase (Sigma-Aldrich, 10 nM in MP Buffer; 56, 112 and 224 kDa) and Thyroglobulin (6 nM in MP Buffer, Sigma-Aldrich, 670 kDa). All measurements were performed at RT.

Native gel electrophoresis

PRC2 and refolded G4 RNA were incubated as indicated in RNP assembly. Immediately after, glutaraldehyde was added at final concentration of 0.1% and reactions were incubated on ice for 25 min prior to quenching with 25 mM Tris (pH 7.5 at RT). For reaction of 600 nM PRC2 and 100 nM RNA, 35 µl was directly loaded to 1% agarose gel (SeaKem GTG Agarose). For reaction of 1 µM PRC2 and 0.5 µM RNA, sample was diluted after quenching to 200 nM PRC2 and 100 nM RNA with RNP complex buffer, then 35 µl was loaded. Gel ran at 66 V for 90 min in a 4°C cold room. The gel was stained with 1X SYBR Gold Nucleic Acid Gel Stain (Invitrogen) for 20 min, and image was collected by GelDoc Go Imaging System (Bio-Rad).

Microscale thermophoresis (MST)

3'-Cy5-labeled RNA oligos, 1G4 and $(GA)_{20}$, were purchased from IDT RNA oligonucleotide synthesis including HPLC purification service. RNA oligos were heated, snap-cooled, and refolded as indicated in RNP assembly. Our MST instrument is a Nano-BLUE/RED Monolith

NT.115 from NanoTemper Technologies, equipped with two LED-filter combinations. We followed manufacturer's instructions for our experiments. Briefly, 20 nM RNA with serial dilutions of PRC2 proteins were incubated in MST assay buffer (10 mM HEPES pH 7.9 at 4°C, 50 mM KCl, 2 mM $MgCl_2$, 0.5 mg/ml BSA, and 1mM TCEP) at 30°C for 15 min prior to measurement. Lithium chloride MST assay buffer (10 mM HEPES pH 7.9 at 4°C, 50 mM LiCl, 2 mM $MgCl_2$, 0.5 mg/ml BSA, and 1mM TCEP) was substituted in particular reactions. The graphic plots were generated from the default evaluation software provided by the equipment.

Fluorescence polarization (FP)

Regular FP assays (binding affinity measurement) and modified FP assays (competitive dissociation measurement) were performed as previously described (*20*).

For FP binding assays, pre-reaction mix was prepared with 5 nM RNA or DNA in binding buffer (50 mM TRIS pH 7.5 at 25°C, 10 or 25 mM KCl, 2.5 mM $MgCl_2$, 0.1 mM $ZnCl_2$, 0.1 mg/mL BSA, 5% glycerol, and 2 mM 2-mercaptoethanol), then dispensed in 36 µL volumes into the wells of a 384-well black microplate (Corning #3575). Six-subunit PRC2 was prepared at 10X the reported concentrations via serial dilution in binding buffer. Binding reactions were initiated by addition of 4 µL of PRC2 solution to the corresponding pre-reaction mix, then incubated 30 min at room temperature. Fluorescence polarization readings were then taken for 30 min in 30 s intervals with a TECAN Spark microplate reader (Ex. = 481 ± 20 nm, Em. = 526 ± 20 nm). Each experiment had 2 or 4 technical replicates per protein concentration (as indicated). Raw data were analyzed in R v4.1.1 with the FPalyze function (FPalyze v1.3.1 package; github.com/whemphil/FPalyze). Briefly, polarization versus time data were calculated for each reaction, the last 10 data points for each reaction were averaged to generate an equilibrium polarization value, and equilibrium polarization values were plotted as a function of protein concentration. Plot data were regressed with Eq. 1.1-2 to calculate K_d^{app} (apparent equilibrium dissociation constant) and n (Hill coefficient) for the interactions. Values from regression with Eq. 1.1 (non-Hill regression) are reported in Fig. 3f.

In the modified FP assays, pre-reaction mix was prepared with 5 nM RNA and 100 nM PRC2 in binding buffer (50 mM TRIS pH 7.5 at 25°C, 10 mM KCl, 2.5 mM $MgCl_2$, 0.1 mM $ZnCl_2$, 0.1 mg/mL BSA, 5% glycerol, and 2 mM 2-mercaptoethanol), then dispensed in 36 µL volumes into the wells of a 384-well black microplate (Corning #3575). Decoy dsDNA was prepared at 10X the reported concentrations via serial dilution in binding buffer. Pre-reaction mix and decoy dilutions were then incubated at 25°C for 30 min to attain thermal and binding equilibrium. Competitive dissociation reactions were initiated by addition of 4 µL of the respective decoy concentration to the corresponding pre-reaction mix, then fluorescence polarization readings were immediately (the delay between initiation of the first reactions and the first polarization reading was ~90 s) taken at 25°C for 120 min in 30 s intervals with a TECAN Spark microplate reader (Ex. = 481 ± 20 nm, Em. = 526 ± 20 nm). Each experiment had 4 technical replicates per decoy concentration. Raw data were analyzed in R v4.1.1 with the FPalyze function (FPalyze v1.3.1 package; github.com/whemphil/FPalyze). Briefly, polarization versus time data were calculated for each reaction, the polarization data were normalized to the maximum and minimum polarization across all reactions, and each normalized reaction was fit with an exponential dissociation function (Eq. 2.1) to determine N_{min}, λ, and k_{off}^{obs} (Eq. 2.2). Then k_{off}^{obs} values were plotted as a function of decoy dsDNA concentration. Plot data were regressed via Eq. 3.1, then Eq. 3.2 with tuning parameters constrained to the Eq. 3.1 solutions, and the

regression models were compared with the Bayesian Information Criterion (BIC). Rate constants for PRC2-RNA dissociation (k_{-1}) and for PRC2 RNA→dsDNA direct transfer (k_{θ}) were reported from the best-performing regression model.

In the equations below, $[E_T]$ is the total PRC2 concentration, FP_E is equilibrium polarization at a given $[E_T]$, FP_{max} is the maximum equilibrium polarization, FP_{min} is the minimum equilibrium polarization, K_d is the apparent equilibrium dissociation constant, n is the Hill coefficient, N_t is relative polarization at a given time (t), N_{min} is the minimum relative polarization, λ is the exponential rate constant, $[D_T]$ is the total decoy dsDNA concentration, k_{off}^{obs} is the apparent rate of complex (PRC2-RNA) dissociation at a specific $[D_T]$, k_{-1} is the first-order rate constant for PRC2-RNA dissociation, k_{θ} is the second-order rate constant for PRC2 direct transfer from RNA to dsDNA, and α and β are arbitrary tuning parameters.

(Eq. 1.1) $$FP_E = (FP_{max} - FP_{min})\frac{[E_T]}{[E_T] + K_d} + FP_{min}$$

(Eq. 1.2) $$FP_E = (FP_{max} - FP_{min})\frac{([E_T])^n}{([E_T])^n + (K_d)^n} + FP_{min}$$

(Eq. 2.1) $$N_t = (1 - N_{min})\, e^{-\lambda t} + N_{min}$$

(Eq. 2.2) $$k_{off}^{obs} = (1 - N_{min})\, \lambda$$

(Eq. 3.1) $$k_{off}^{obs} \approx \frac{[D_T]^\beta}{\alpha^\beta + [D_T]^\beta} k_{-1P} + k_{\theta D}\, [D_T]$$

(Eq. 3.2) $$k_{off}^{obs} \approx \frac{[D_T]^\beta}{\alpha^\beta + [D_T]^\beta} k_{-1P}$$

Methyltransferase activity assay

For reaction time-based assays, 400 nM PRC2 and 400 nM refolded G4 RNAs were pre-incubated at 30°C for 30 min to reach binding equilibrium, and then assembled into methyltransferase reaction mix including 1X methyltransferase buffer (25 mM HEPES pH 7.9 at 4°C, 50 mM KCl, 2 mM $MgCl_2$, 10% glycerol, and 1mM TCEP), 0.1 mg/ml BSA, 1X protease inhibitor, 1 µl/20 µl RNase inhibitor, 10 µM ^{14}C-SAM (PerkinElmer), and 0.16 mg/ml polynucleosome or 200 nM trinucleosome or 250 nM mononucleosome or 6.5 µM recombinant H3 (NEB). Samples were collected at 0, 3, 6, 10, 15, 20, 30, 45, and 60 min from the same tube incubating at 30°C. Proteins were separated through NuPAGE 4-12% gel (Invitrogen) by running at 180V for 52 min. Gel was vacuum dried at 80°C for 30 min, and then exposed to phosphorimaging plates. Signal intensities were quantified by ImageQuant TL and plotted by Microsoft Excel.

For RNA concentration-based assays, 400 nM PRC2 and serial dilutions of refolded 1G4 RNA were pre-incubated and assembled into methyltransferase reaction mix including 1X methyltransferase buffer, 0.1 mg/ml BSA, 1X protease inhibitor, 1 µl/20 µl RNase inhibitor, 10 µM ^{14}C-SAM (PerkinElmer), and 250 nM trinucleosome. Reactions were performed at 30°C for 20 min. NuPAGE running, signal acquisition and quantification were same as previous.

Nucleosome-RNA competition assay

We prepared ^{32}P-labeled trinucleosomes in-house by adding a small fraction of radiolabeled DNA into non-labeled DNA during trinucleosome assembly. 600 nM PRC2, 150 nM labeled trinucleosome, and serial dilutions of refolded 1G4 RNA were combined in RNP complex buffer (25 mM HEPES pH 7.9 at 4°C, 50 mM KCl, 2 mM $MgCl_2$, 10% glycerol, and 1mM TCEP) at

30°C for 30 min. Sample was loaded to 1% agarose gel (SeaKem GTG Agarose) buffered with 1X TBE and resolved at 66 V for 110 min in a 4°C cold room. Gels were vacuum dried for 60 min at 80°C. Dried gels were exposed to phosphorimaging plates and signal acquisition was performed using a Typhoon Trio phosphorimager (GE Healthcare).

Zebrafish morpholino and mRNA rescue injections

Zebrafish were utilized in accordance with approvals from Beth Israel Deaconess Medical Center and Boston Children's Hospital Institutional Animal Care and Use Committees. Zebrafish knockdown was performed with *ezh2* translation-blocking morpholinos (*ezh2*-MO: 5′-CCGATTTCCTCCCGGTCAATCCCAT-3′) (*45*) and standard control MO (5’-CCTCTTACCTCAGTTACAATTTATA-3’) resuspended in nuclease-free water and stored at room temperature (MO, GeneTools). For mRNA rescue experiments, human WT or mutated EZH2 capped mRNAs were synthesized using the mMessage mMachine SP6 transcription kit (Thermo Fisher Scientific) and purified by NucleoSpin RNA Clean-up XS column (MACHEREY-NAGEL). Wildtype TU strain of zebrafish embryos were injected at the 1-cell stage with 4 ng *ezh2*-MO or control-MO alone or in combination with mRNA at 25 ng, 50 ng, or 100ng, as indicated. At 48 hours post fertilization (hpf), embryos were dechorionated and assessed blindly for growth impairment using the ZEISS Axiocam 305 color camera. Growth impairment and viability were monitored up to 72 hpf with $n \geq 50$ embryos/condition from ≥ 3 replicate clutches. Statistical analyses were performed on GraphPad Prism 9. Fisher’s exact test was used to determine the P value and statistical significance by comparing the percentage of normal embryos to the percentage of abnormal embryos (both reduced growth and severely reduced growth).

Supplementary figures and legends

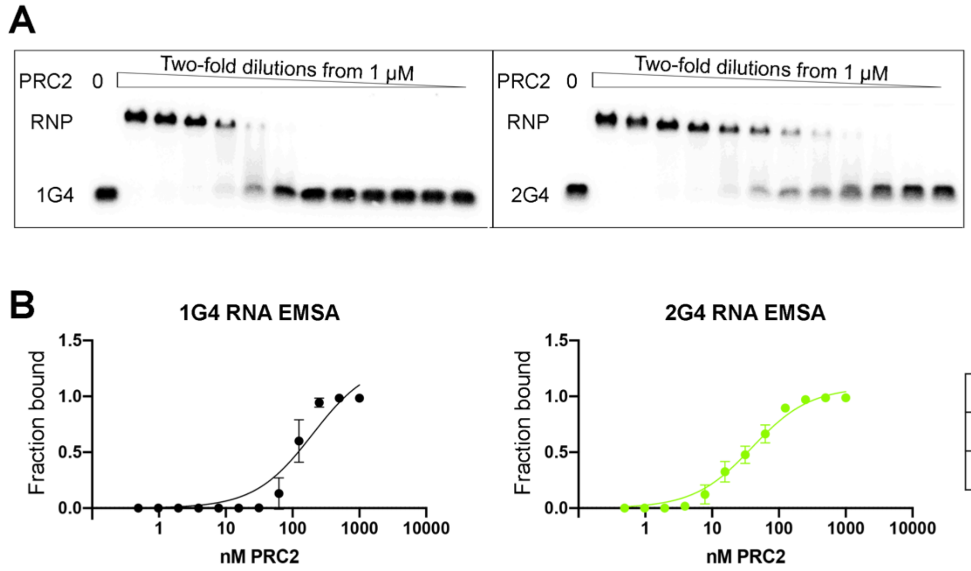

Kd	WT PRC2
1G4	120 ± 43 nM
2G4	32 ± 4 nM

Fig. S1. Six-subunit PRC2 complex binding to G4-forming RNAs in vitro. (**A**) Representative EMSA results of six-subunit PRC2 binding to 1G4 and 2G4. (**B**) Quantification of three EMSA replicates. Error bars indicate standard deviation (SD) of three replicates performed on different days.

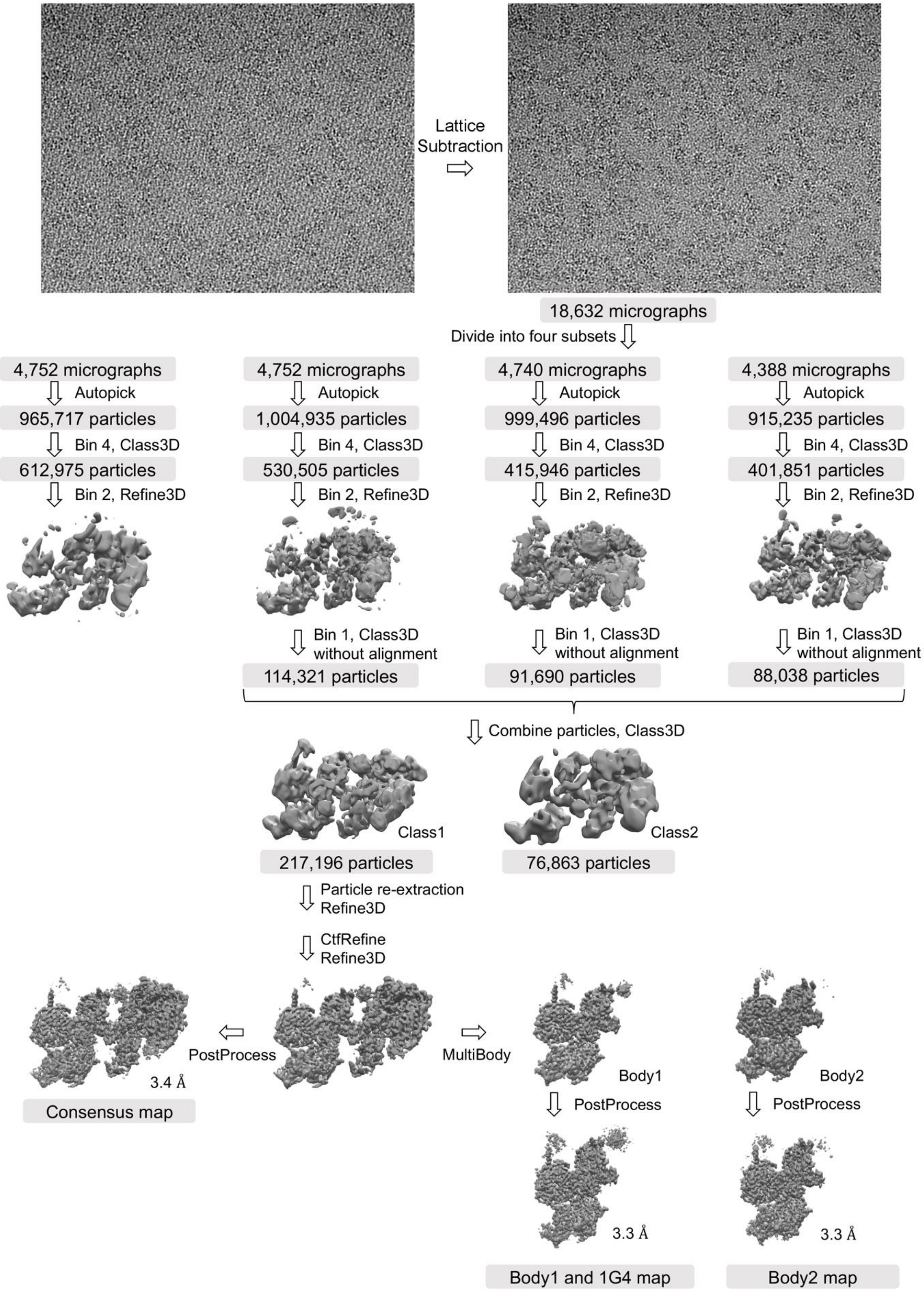

Fig. S2. Single-particle cryo-EM image processing workflows for PRC2-1G4 RNA complex.

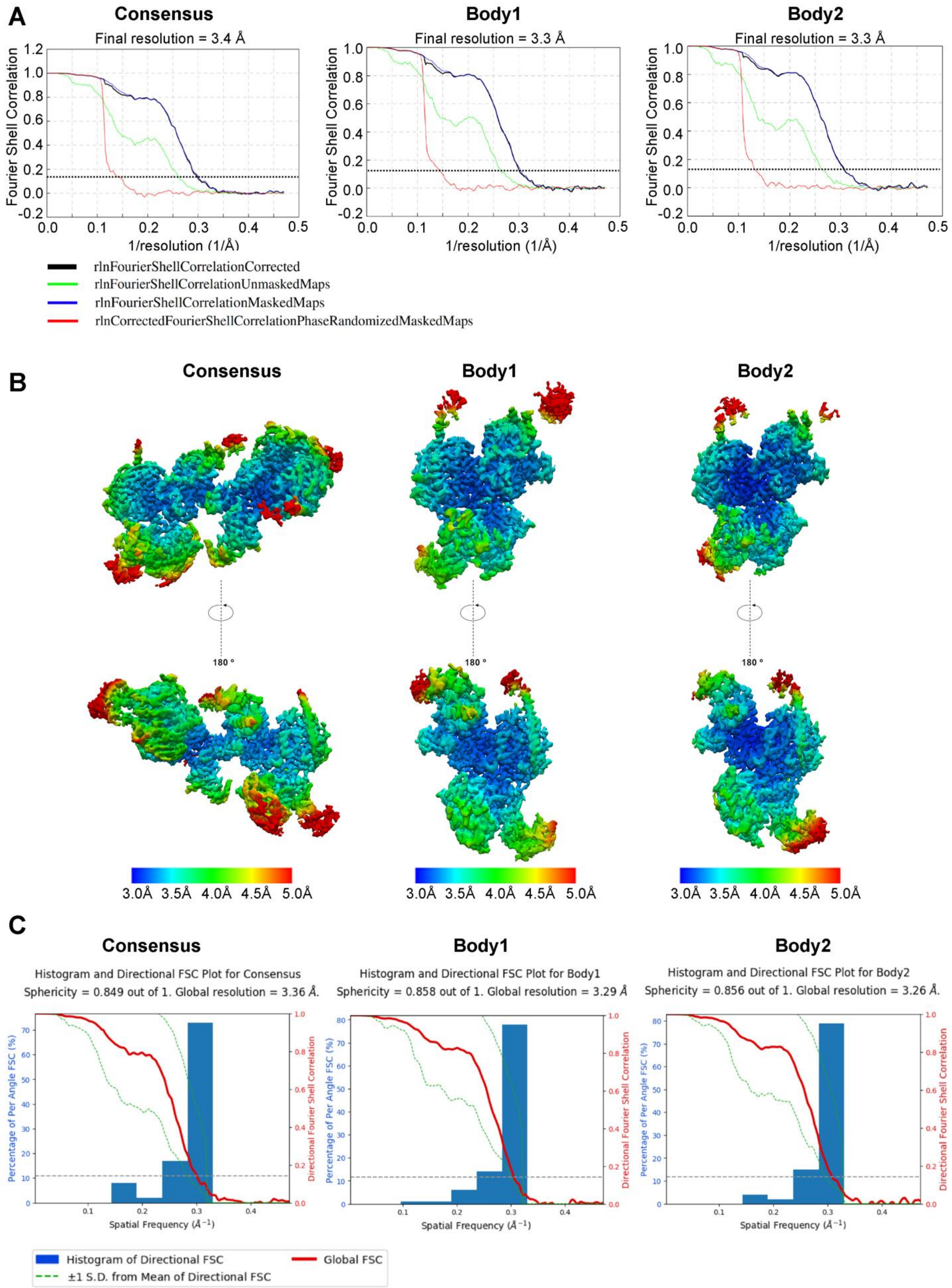

A
Consensus
Final resolution = 3.4 Å
Body1
Final resolution = 3.3 Å
Body2
Final resolution = 3.3 Å
Fourier Shell Correlation
1/resolution (1/Å)
rlnFourierShellCorrelationCorrected
rlnFourierShellCorrelationUnmaskedMaps
rlnFourierShellCorrelationMaskedMaps
rlnCorrectedFourierShellCorrelationPhaseRandomizedMaskedMaps
B
Consensus
Body1
Body2
180 °
3.0Å 3.5Å 4.0Å 4.5Å 5.0Å
C
Consensus
Histogram and Directional FSC Plot for Consensus
Sphericity = 0.849 out of 1. Global resolution = 3.36 Å.
Body1
Histogram and Directional FSC Plot for Body1
Sphericity = 0.858 out of 1. Global resolution = 3.29 Å
Body2
Histogram and Directional FSC Plot for Body2
Sphericity = 0.856 out of 1. Global resolution = 3.26 Å.
Percentage of Per Angle FSC (%)
Directional Fourier Shell Correlation
Spatial Frequency (Å⁻¹)
Histogram of Directional FSC
±1 S.D. from Mean of Directional FSC
Global FSC

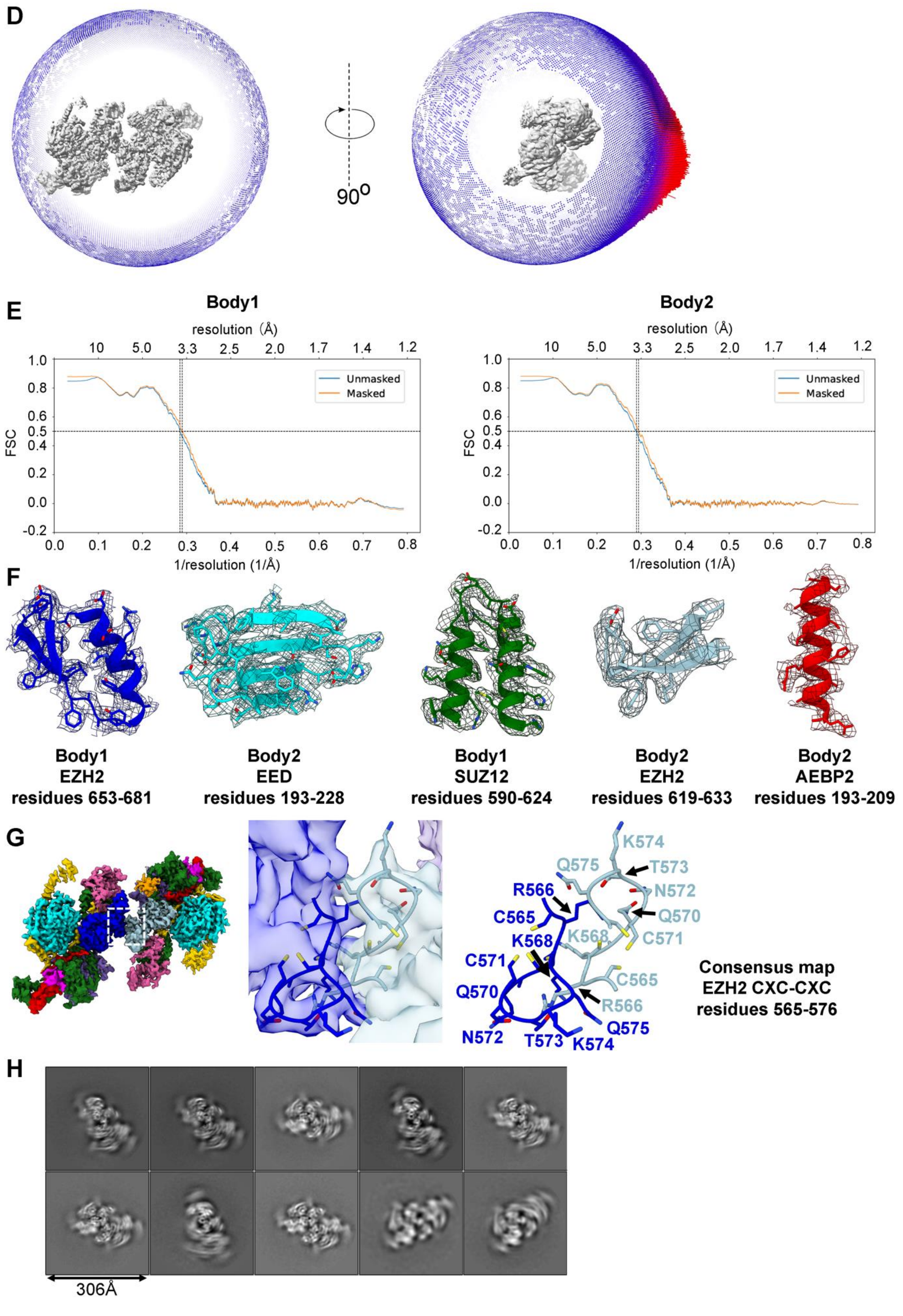

D
90°
E
Body1
Body2
resolution (Å)
10 5.0 3.3 2.5 2.0 1.7 1.4 1.2
FSC
1/resolution (1/Å)
Unmasked
Masked
F
Body1
EZH2
residues 653-681
Body2
EED
residues 193-228
Body1
SUZ12
residues 590-624
Body2
EZH2
residues 619-633
Body2
AEBP2
residues 193-209
G
K574
Q575
T573
N572
R566
Q570
C565
K568
C571
C571
C565
Q570
R566
Q575
N572
T573
K574
Consensus map
EZH2 CXC-CXC
residues 565-576
H
306Å

Fig. S3. Cryo-EM map analysis. (**A**) Fourier shell correlation (FSC) curves of consensus map and maps of two individual bodies from multibody refinement. 0.143 intercepts are indicated by dashed lines. (**B**) Local-resolution density maps of consensus and two bodies. (**C**) 3D FSC of consensus map and two bodies. (**D**) Euler angle distribution for the particles after consensus map refinement. (**E**) Model vs Map FSC for Phenix-refined models of two bodies. (**F**) Examples of cryo-EM density from multibody refinement maps (contour level: 0.015) and built-in models. (**G**) Cryo-EM density of the CXC-CXC dimerization interface from the consensus map (contour level: 0.016) and built-in models. Dashed box highlights the region zoomed in the next panel. Another view of the same region is shown in (Fig. 3A). (**H**) A subset of 2D class averages from the final 217,196 particles used to generate the consensus map.

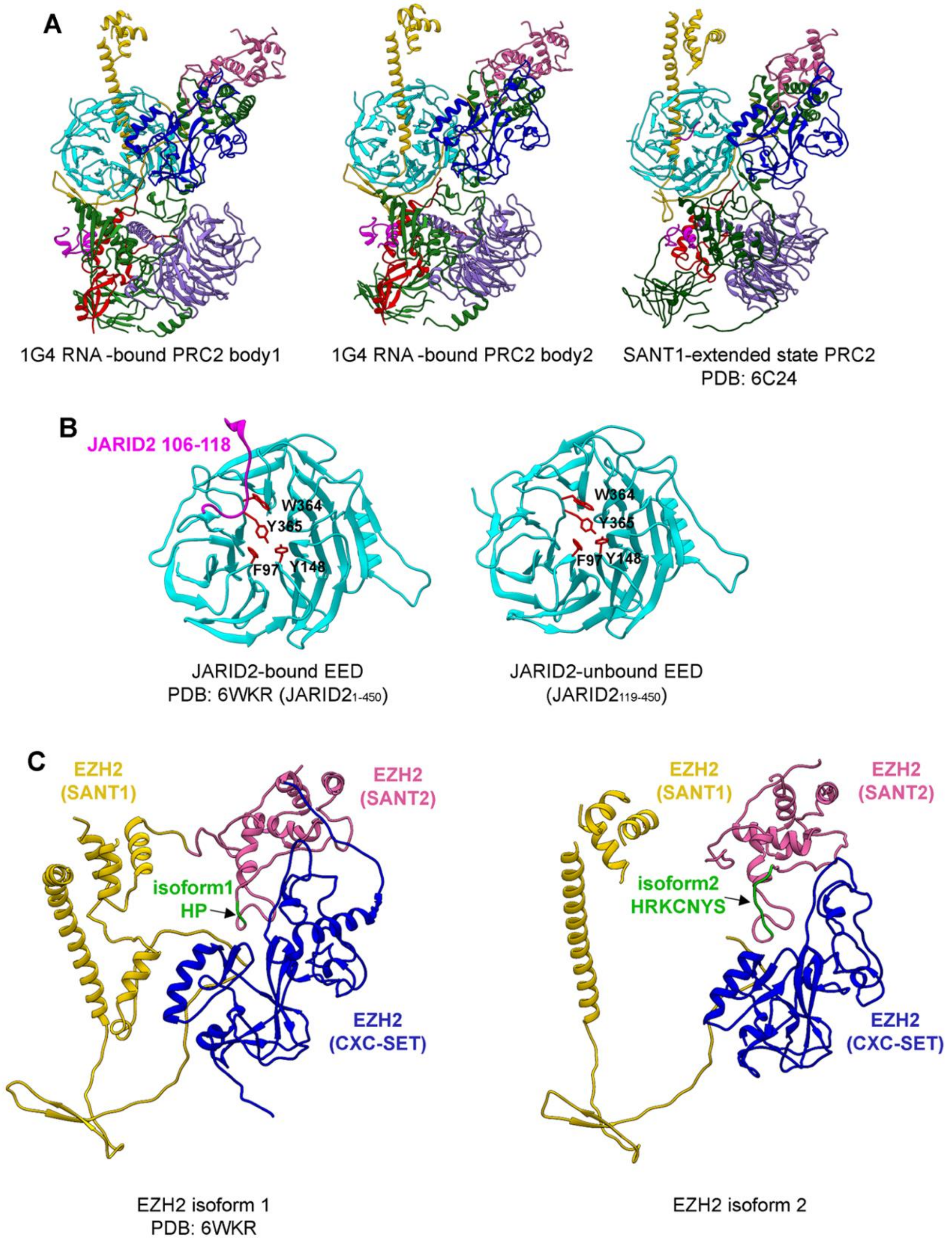

Fig. S4. Structure comparisons. (**A**) Overall subunit arrangements of RNA-bound PRC2 body1, body2 and SANT1-extended state PRC2 are nearly identical. EZH2 (SANT1) is highlighted in gold, EZH2 (SANT2) in hot pink, EZH2 (CXC-SET) in blue, EED in cyan, RBAP48 in purple, SUZ12 in green, JARID2 in magenta, and AEBP2 in red. (**B**) Structure comparison of JARID2-bound EED and JARID2-free EED. We truncated JARID2 N-terminus (1-118) to avoid JARID2-RNA competition suggested by others (*25*). The N-terminal region of JARID2 contains an EED binding site (residues 106-118 with K116me3) functioning as an

H3K27me3 mimic and PRC2 activator (*49*). Although our $JARID2_{119-450}$ lacks it, EED does not experience any major conformational change. We used $JARID2_{119-450}$ without additional C-terminal truncation to be consistent with the cryo-EM structure of the PRC2-nucleosome complex (*27*). EED and JARID2 are colored cyan and magenta, respectively. EED residues F97, Y148, W364, and Y365 which have been proposed for JARID2 association are shown in stick representation (red). (**C**) Structure comparison of EZH2 isoform 1 and isoform 2 (297-298:HP→HRKCNYS) showing the same secondary and tertiary arrangements. EZH2 isoform 1 structure is adapted from nucleosome-bound PRC2 (PDB:6WKR). Isoform 2 structure is modeled from RNA-bound PRC2. SANT1 domain is highlighted in gold, SANT2 in hot pink, CXC-SET in blue, and the different residues between isoforms in green.

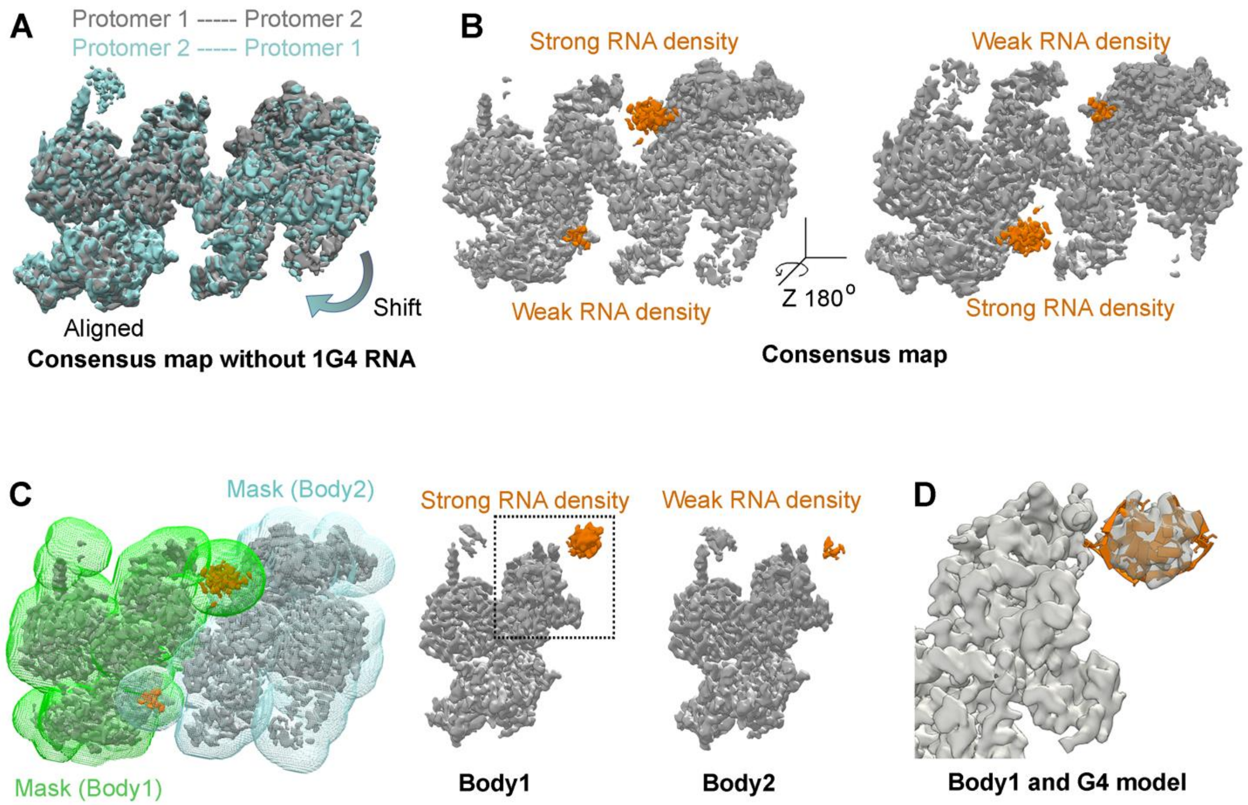

Fig. S5. PRC2-1G4 RNP has imperfect C_2 symmetry. (**A**) The consensus map without 1G4 density was duplicated and rotated 180 degrees cross the Z-axis. The protomer 1 of original map (gray) is aligned with the protomer 2 of the rotated map (cyan) to highlight the imperfect symmetry. (**B**) A strong RNA density and a weak density on two symmetric sites were distinguished from the consensus map. (**C**) Left: We grouped one PRC2 promoter with strong RNA density into Body1 and the other promoter with weak density into Body2 in the process of multibody refinement. Two masks are highlighted in green and blue, respectively. Right: Refinement maps of Body1 and Body2 (contour level: 0.015, hide dust size 5). Highlighted area is zoomed in (D). (**D**) G4-structure model (adapted from PDB:2M18) was fitted into the strong RNA density of Body1 (contour level: 0.015, hide dust size 5). We did not obtain high-quality RNA density for de novo modeling, but the size of the density represents a single G4 RNA.

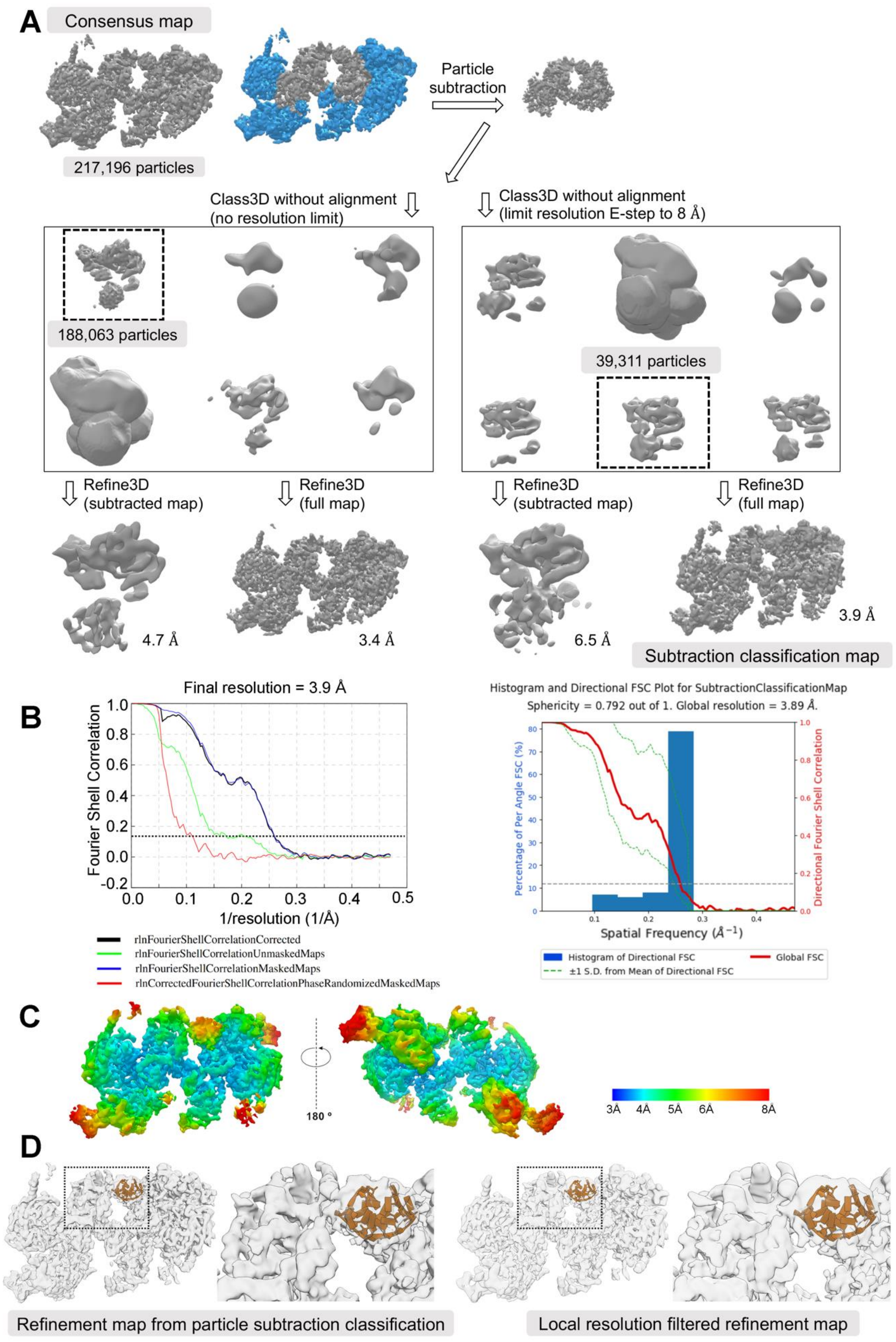
A
Consensus map
217,196 particles
Particle subtraction
Class3D without alignment
(no resolution limit)
188,063 particles
Class3D without alignment
(limit resolution E-step to 8 Å)
39,311 particles
Refine3D
(subtracted map)
Refine3D
(full map)
4.7 Å
3.4 Å
6.5 Å
3.9 Å
Subtraction classification map
B
Final resolution = 3.9 Å
Fourier Shell Correlation
1/resolution (1/Å)
rlnFourierShellCorrelationCorrected
rlnFourierShellCorrelationUnmaskedMaps
rlnFourierShellCorrelationMaskedMaps
rlnCorrectedFourierShellCorrelationPhaseRandomizedMaskedMaps
Histogram and Directional FSC Plot for SubtractionClassificationMap
Sphericity = 0.792 out of 1. Global resolution = 3.89 Å.
Percentage of Per Angle FSC (%)
Directional Fourier Shell Correlation
Spatial Frequency (Å^-1)
Histogram of Directional FSC
Global FSC
±1 S.D. from Mean of Directional FSC
C
180 °
3Å 4Å 5Å 6Å 8Å
D
Refinement map from particle subtraction classification
Local resolution filtered refinement map

Fig. S6. Particle subtraction classification provides a 3.9 Å-resolution map revealing more information on PRC2-G4 interactions. (**A**) Processing workflows of particle subtraction and classification of G4 RNA plus neighboring EZH2 SET and SANT2 regions. We started with the consensus map to exclude regions as indicated in blue color. Classification without alignment of the subtracted particles were carried out with a regularization parameter T = 16 either by limiting resolution to 8 Å or by using all information till Nyquist. Limiting resolution produced better classification results with stronger density for RNA and interacting regions likely due to the high degree of conformational flexibility in this region. The selected classes, highlighted in boxes, were used for further refinement. (**B**) FSC and 3D FSC curves of the subtraction classification map. (**C**) Local-resolution density maps of the subtraction classification map. (**D**) 3D refinement and local resolution filtered map after particle subtraction and classification (contour level: 0.014, hide dust size 5) show that the identified strong density is similar in size to a G4 RNA model and can indeed fit G4-structure model (PDB:2M18; orange).

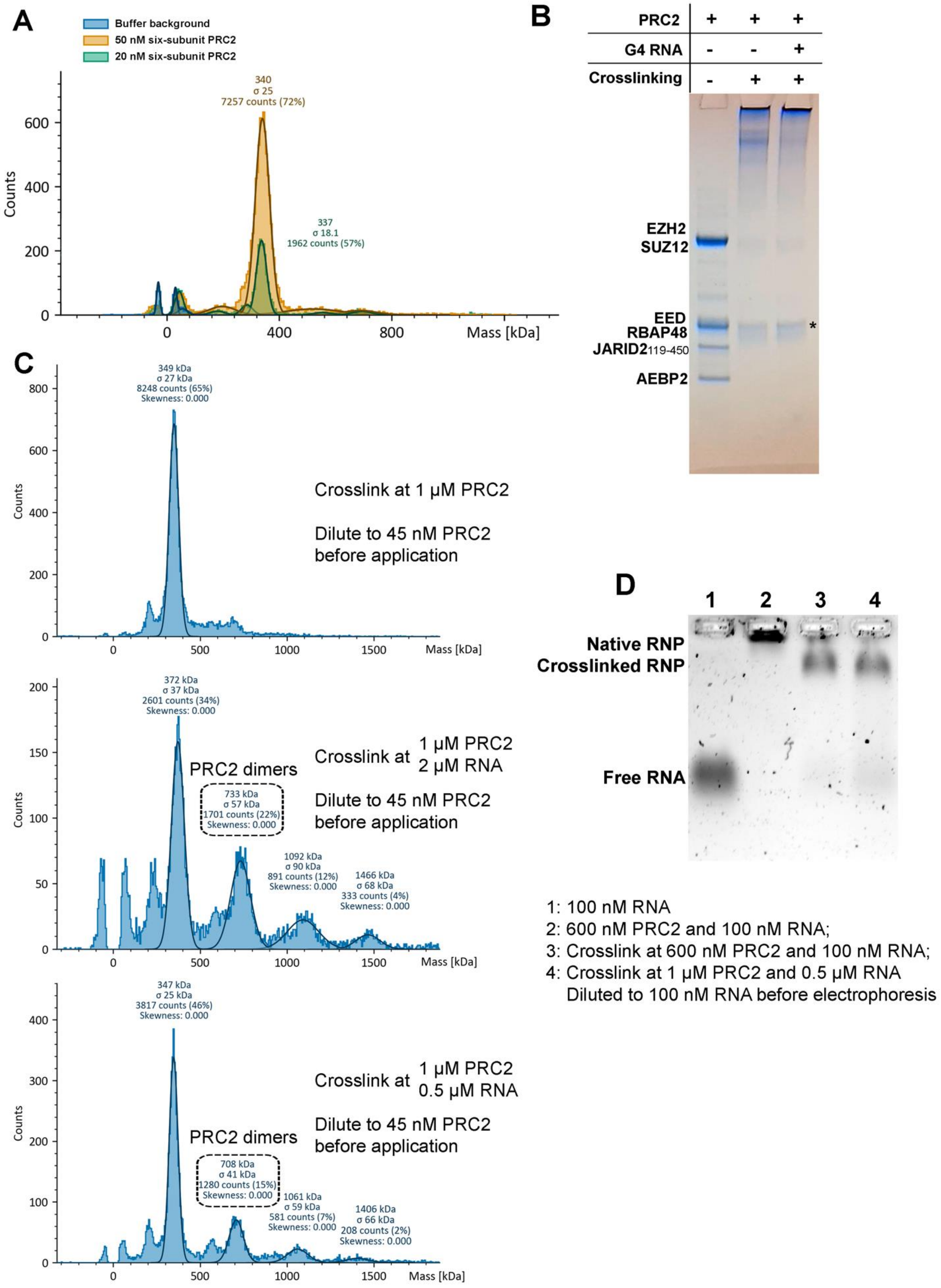
A
Buffer background
50 nM six-subunit PRC2
20 nM six-subunit PRC2
340
σ 25
7257 counts (72%)
337
σ 18.1
1962 counts (57%)
Counts
Mass [kDa]
B
PRC2
G4 RNA
Crosslinking
EZH2
SUZ12
EED
RBAP48
JARID2119-450
AEBP2
*
C
349 kDa
σ 27 kDa
8248 counts (65%)
Skewness: 0.000
Crosslink at 1 μM PRC2
Dilute to 45 nM PRC2 before application
372 kDa
σ 37 kDa
2601 counts (34%)
Skewness: 0.000
PRC2 dimers
733 kDa
σ 57 kDa
1701 counts (22%)
Skewness: 0.000
Crosslink at 1 μM PRC2 2 μM RNA
Dilute to 45 nM PRC2 before application
1092 kDa
σ 90 kDa
891 counts (12%)
Skewness: 0.000
1466 kDa
σ 68 kDa
333 counts (4%)
Skewness: 0.000
347 kDa
σ 25 kDa
3817 counts (46%)
Skewness: 0.000
Crosslink at 1 μM PRC2 0.5 μM RNA
Dilute to 45 nM PRC2 before application
PRC2 dimers
708 kDa
σ 41 kDa
1280 counts (15%)
Skewness: 0.000
1061 kDa
σ 59 kDa
581 counts (7%)
Skewness: 0.000
1406 kDa
σ 66 kDa
208 counts (2%)
Skewness: 0.000
D
1 2 3 4
Native RNP
Crosslinked RNP
Free RNA
1: 100 nM RNA
2: 600 nM PRC2 and 100 nM RNA;
3: Crosslink at 600 nM PRC2 and 100 nM RNA;
4: Crosslink at 1 μM PRC2 and 0.5 μM RNA
Diluted to 100 nM RNA before electrophoresis

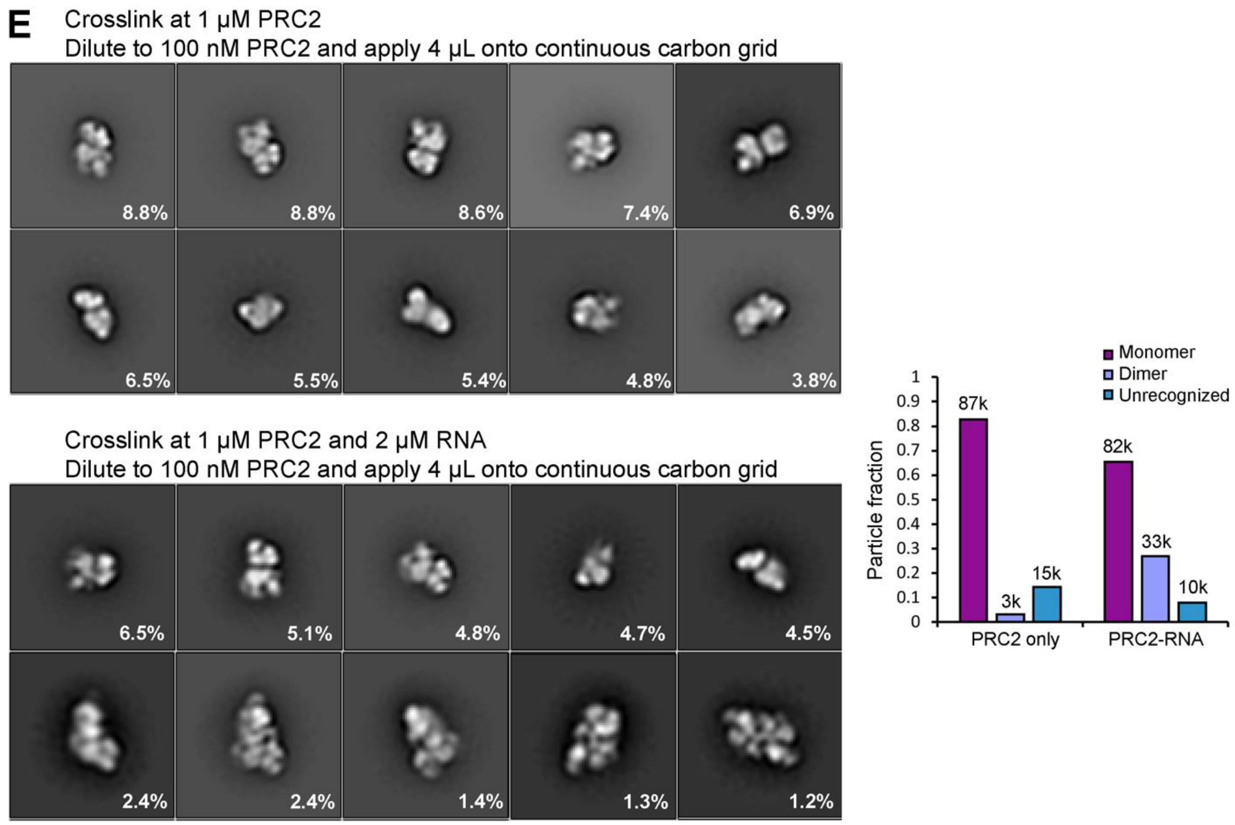

Fig. S7. PRC2 six-subunit complex is a monomer in solution and dimerizes by G4 RNA binding. (**A**) Mass photometry of six-subunit PRC2 at two protein concentrations. (**B**) Coomassie-stained SDS-PAGE gel of PRC2 crosslinked by 0.1% glutaraldehyde. (*), RBAP48 and EED contain WD40 repeat domains that were incompletely crosslinked. (**C**) Mass photometry of crosslinked PRC2 and PRC2-2G4 complexes. Due to technical limitations of mass photometry, the sample concentration is restricted to under 50 nM which is not sufficient to assemble a detectable amount of PRC2-RNA dimers (Kd_{1G4}=120 nM and Kd_{2G4}=32 nM, Fig. S1). Therefore, we crosslinked our reactions at 1 µM PRC2 concentration and diluted to 45 nM before mass photometry. Protein sample (top) has a dominant peak at 349 KDa which constitutes 64% of counts with minor contaminating peak at approximately 200 kDa, and some non-specifically crosslinked complexes between 400 kDa and 700 kDa. Including 2G4 RNA at two different concentrations (middle and bottom) led to a reduction of monomeric PRC2 and the appearance of a second-most abundant peak at approximately 720 kDa. Two additional peaks corresponded to complexes with three or four PRC2, respectively, which were not detected in native PRC2-RNA complexes by size-exclusion chromatography or EM. (**D**) Native gel electrophoresis of crosslinked PRC2-RNA complexes visualizing the SYBR Gold stained RNA. This result indicates that glutaraldehyde crosslinking did not dissociate RNA from PRC2, although the crosslinked complexes had slightly faster electrophoresis migration than the native complex. (**E**) Left: Representative 2D class averages of crosslinked PRC2 pre-incubated with or without RNA from continuous carbon grids. Fractions of each class are highlighted at the bottom-right (70 classes in total). The presented class averages were collected from the initial 2D classification after particle extraction, which led to lower image quality but unbiased classification of all identified particles from every micrograph. Right: Monomer and dimer particles were quantified. This result is consistent with mass photometry data.

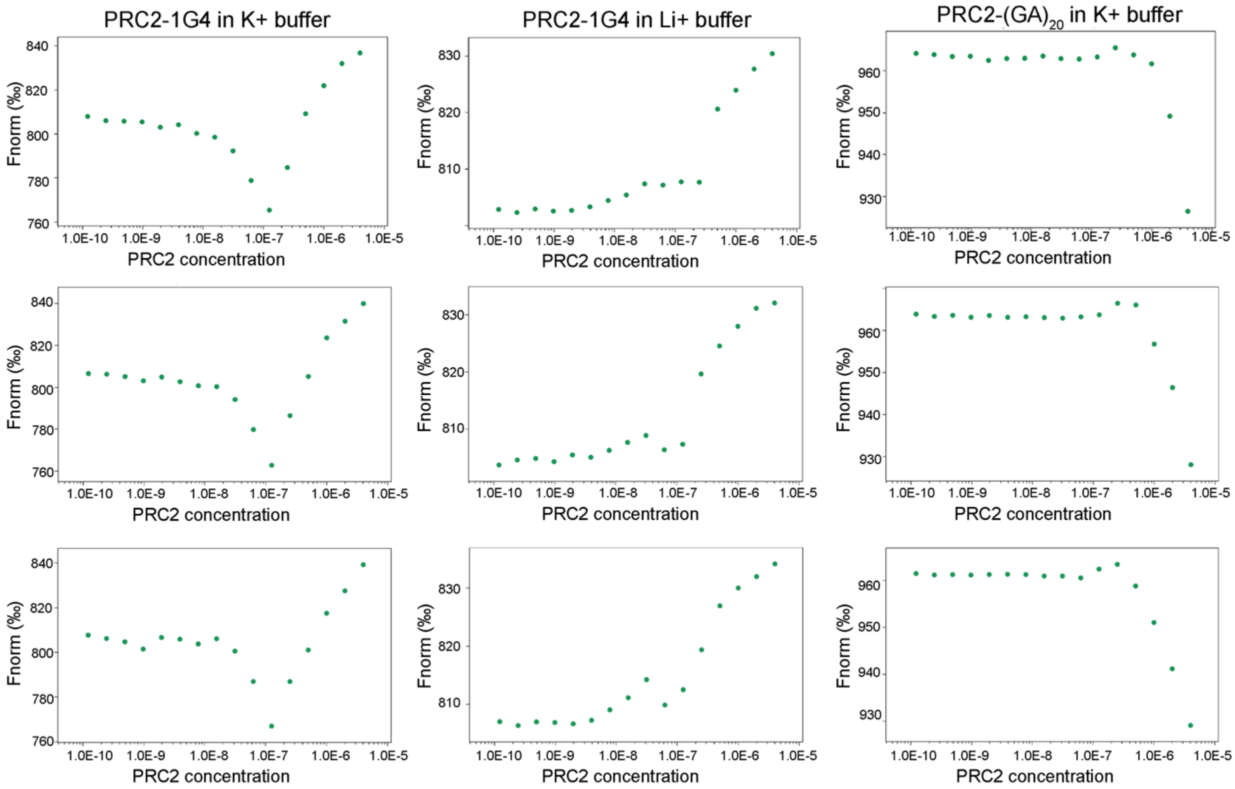

Fig. S8. MST profiles of PRC2-RNA interactions. Microscale thermophoresis (MST) measures the motion of molecules along microscopic temperature gradients that changes upon ligand binding. The fluorescence change in MST signal is normalized (Fnorm), defined as F_{hot}/F_{cold} (F_{hot} as the hot region at 20 s after IR laser heating and F_{cold} as the cold region at 0 s) (*66*). Left panels, three independent replicates of assays carried out in the same K^+ buffer as used in the cryo-EM. Typically, an MST plot of a single-binding event exhibits a canonical monophasic curve and plateau at high concentrations (*67*). Here, PRC2-1G4 binding showed a biphasic curve in which initial data points (PRC2 concentrations <100 nM) represent the first binding event (1 PRC2: 1 RNA) and change to a different MST profile at higher PRC2 concentrations (>100 nM, second binding event, 2 PRC2: 1 RNA). Eventually points are assumed to plateau followed the second binding event, but we could not obtain enough PRC2 to reach saturation. Middle panels, Li^+ destabilizes G4 structures (*68*) serving as a negative control (middle). Therefore, most of data points followed the trend of a monophasic curve. Right panels, $(GA)_{20}$ weakly interacts with PRC2 and also serves a negative control. Therefore, only at very high PRC2 concentrations, reactions started to provide an MST signal separate from the baseline. The current analysis software does not provide statistical analysis for multiple replicates of the biphasic curve, so we present three independent experiments to indicate the reproducibility of these measurements.

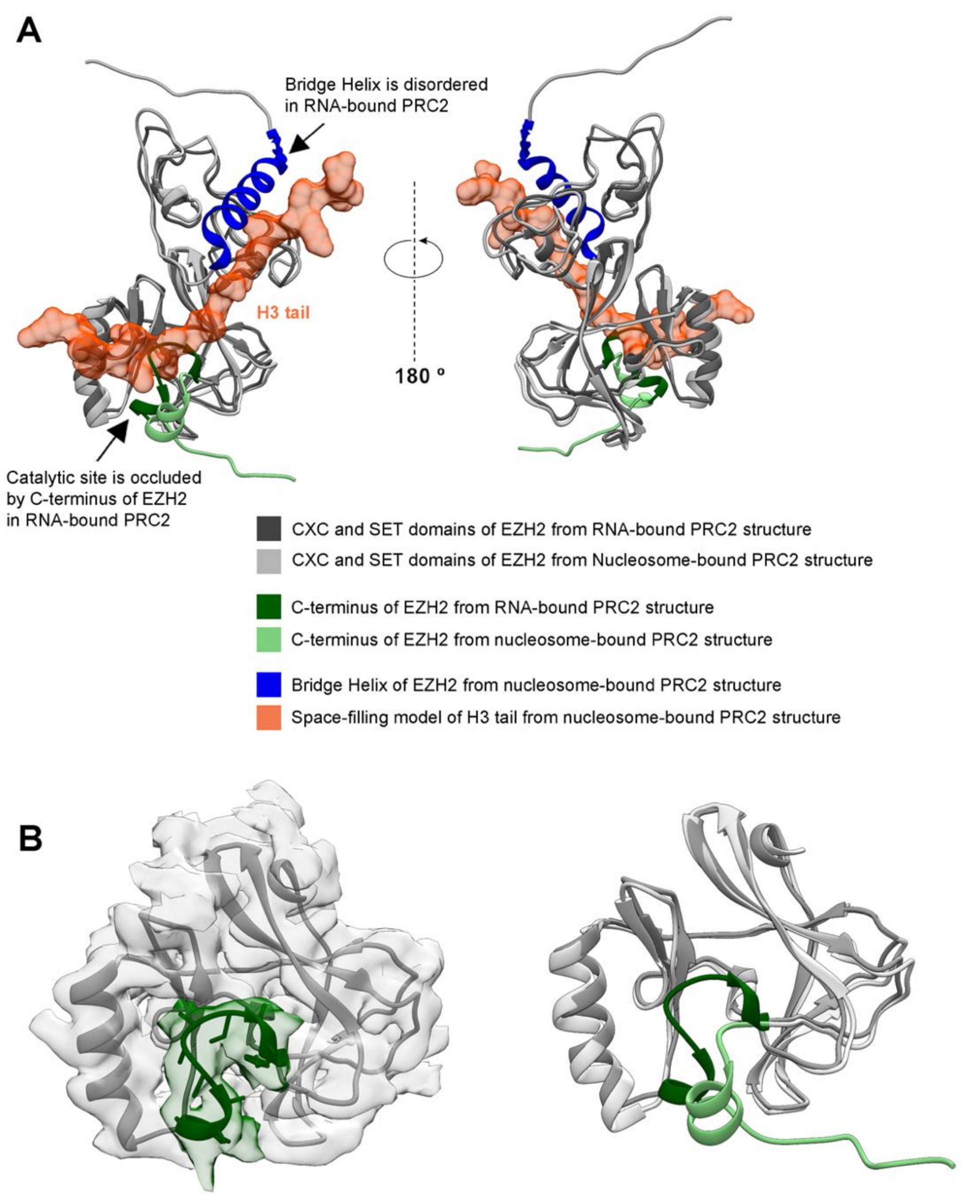

Fig. S9. Comparison of EZH2 CXC and SET domains of nucleosome-bound and RNA-bound PRC2. (**A**) Model of EZH2 CXC and SET domains from 1G4 RNA-bound PRC2 is superimposed onto the nucleosome-bound PRC2 structure (PBD:6WKR) to identify confirmational changes. We identified two significant differences highlighted by arrows. First, Bridge Helix of nucleosome-bound PRC2 is disordered in RNA-bound structure, consistent with its function specifically for DNA and H3 tail binding. Second, C-terminus of EZH2 from RNA-bound PRC2 clashes with H3 tail model obtained from nucleosome-bound PRC2 structure, which suggests a mechanism for RNA-based PRC2 inactivation additionally to CXC domain dimerization. (**B**) Left: The density map and fitted model of the new conformation of the C-terminus of EZH2 in RNA-bound PRC2. C-terminal residues are colored in dark green. Right: Superimposed EZH2 models of 1G4-bound and nucleosome-bound PRC2 at the same view. The C-terminal residues of EHZ2 (SET) in nucleosome bound state is shown as light green to highlight the difference in conformation of this region. Color scheme is identical to panel A.

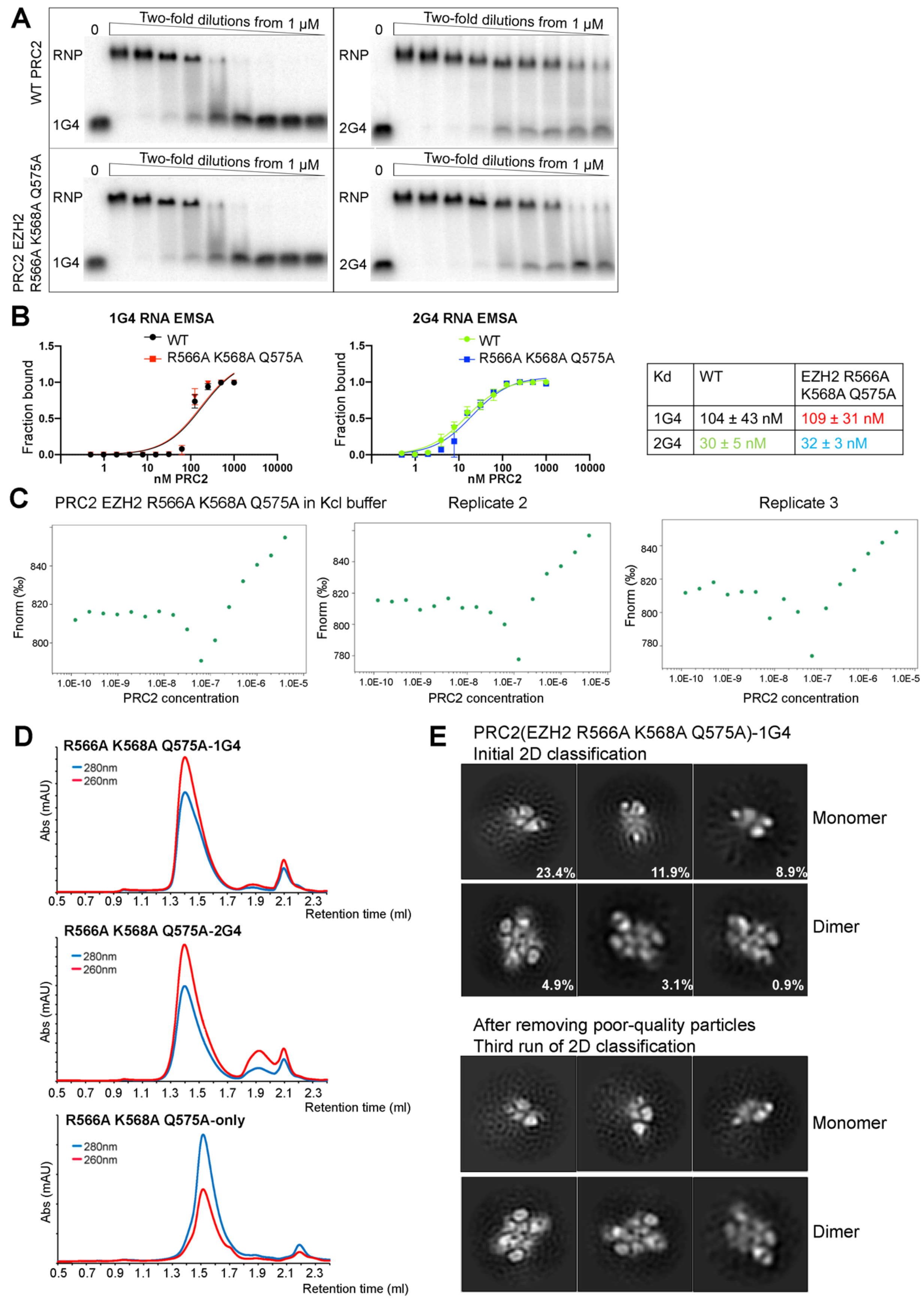

Kd	WT	EZH2 R566A K568A Q575A
1G4	104 ± 43 nM	109 ± 31 nM
2G4	30 ± 5 nM	32 ± 3 nM

Fig. S10. EZH2 R566A K568A Q575A does not impact 1G4-binding and PRC2 dimerization at high concentration. (**A**) Representative EMSA gels of WT PRC2 and EZH2 R566A K568A Q575A binding to 1G4 and 2G4. (**B**) Quantification of three EMSA replicates. Error bars indicate SD of three replicates performed on different days. (**C**) MST data of EZH2 R566A K568A Q575A binding 1G4 RNA. EZH2 R566A K568A Q575A provided the same biphasic curve as WT PRC2 (fig. S7 left), indicating its dimerization potential in high concentration. We present three independent experiments to indicate the reproducibility of this measurement. (**D**) Size-exclusion chromatography of EZH2 R566A K568A Q575A preincubated with 1G4, 2G4 and mock. (**E**) Top: representative 2D-class averages of EZH2 R566A K568A Q575A-1G4 RNA complex collected from streptavidin-affinity grid. Fractions of each class are highlighted at the bottom-right (70 classes in total). The presented class averages were collected from the initial 2D classification immediately after particle extraction, which led to lower image quality but unbiased classification of all identified particles from every micrograph. Bottom: representative class averages of the subsequent 2D classification from the same collection. Image quality of each class was increased by removing poor-quality particles.

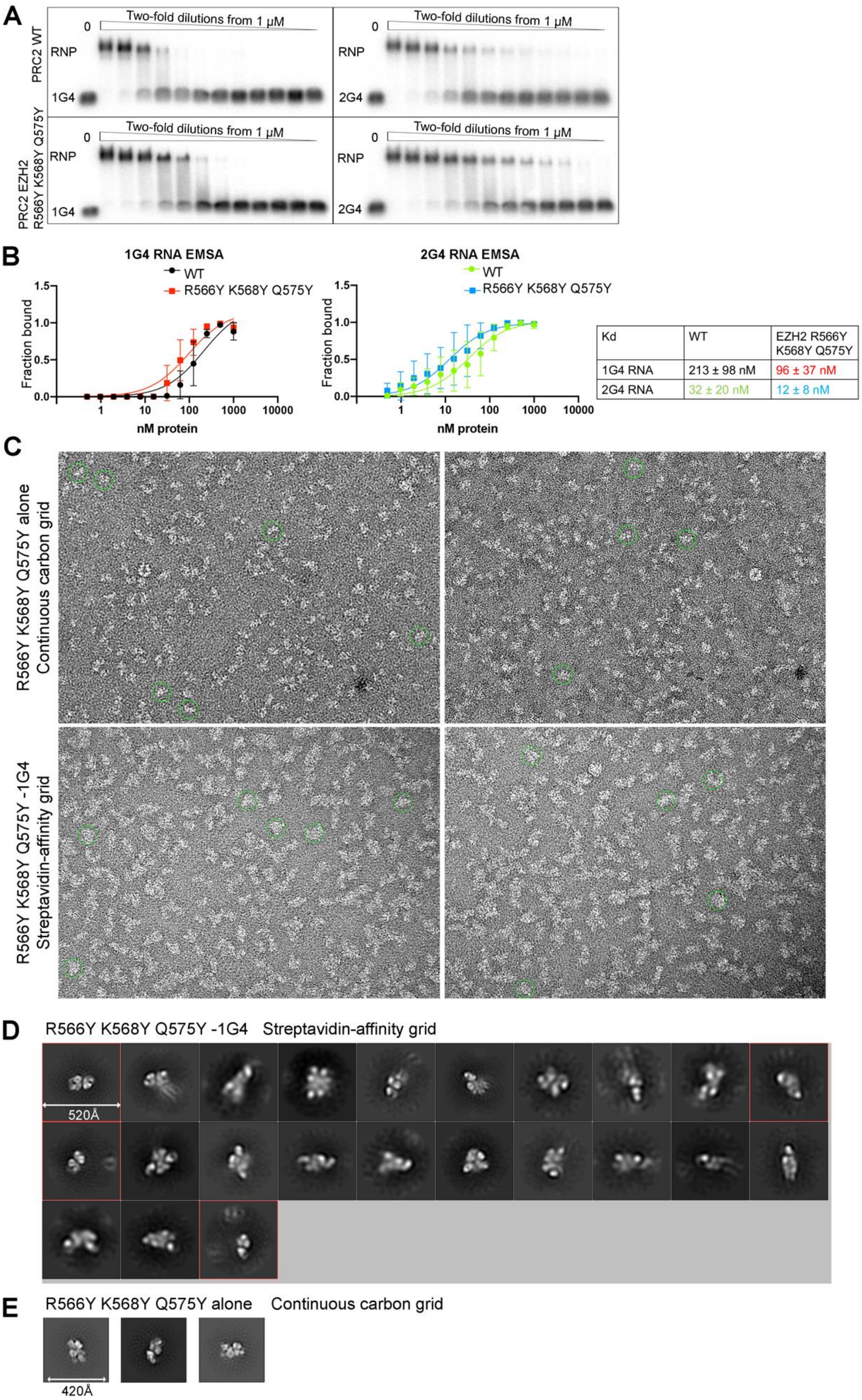

Kd	WT	EZH2 R566Y K568Y Q575Y
1G4 RNA	213 ± 98 nM	96 ± 37 nM
2G4 RNA	32 ± 20 nM	12 ± 8 nM

Fig. S11. EZH2 R566Y K568Y Q575Y mutant of PRC2 increases RNA binding. (**A**) Representative EMSA results of WT PRC2 and EZH2 R566Y K568Y Q575Y binding to 1G4 and 2G4. (**B**) Quantification of three EMSA replicates. Error bars indicate SD of three replicates performed on different days. (**C**) Negative staining EM images of EZH2 R566Y K568Y Q575Y on continuous carbon grid and 1G4-bound RNP on streptavidin-affinity grid after lattice-subtraction. Green circles have a diameter of 250Å to highlight size-difference between monomers and dimers. (**D**) 2D class averages from 196,006 particles of EZH2 R566Y K568Y Q575Y-1G4 collected from streptavidin-affinity grid. EZH2 R566Y K568Y Q575Y may utilize the stacking interactions of aromatic sidechains of tyrosine to form a more flexible CXC-CXC interface. We could identify 4 monomer classes (red circles) and 19 dimer classes (> 70% of particles). (**E**) Examples of 2D class averages from EZH2 R566Y K568Y Q575Y alone. No PRC2 dimer class was identified.

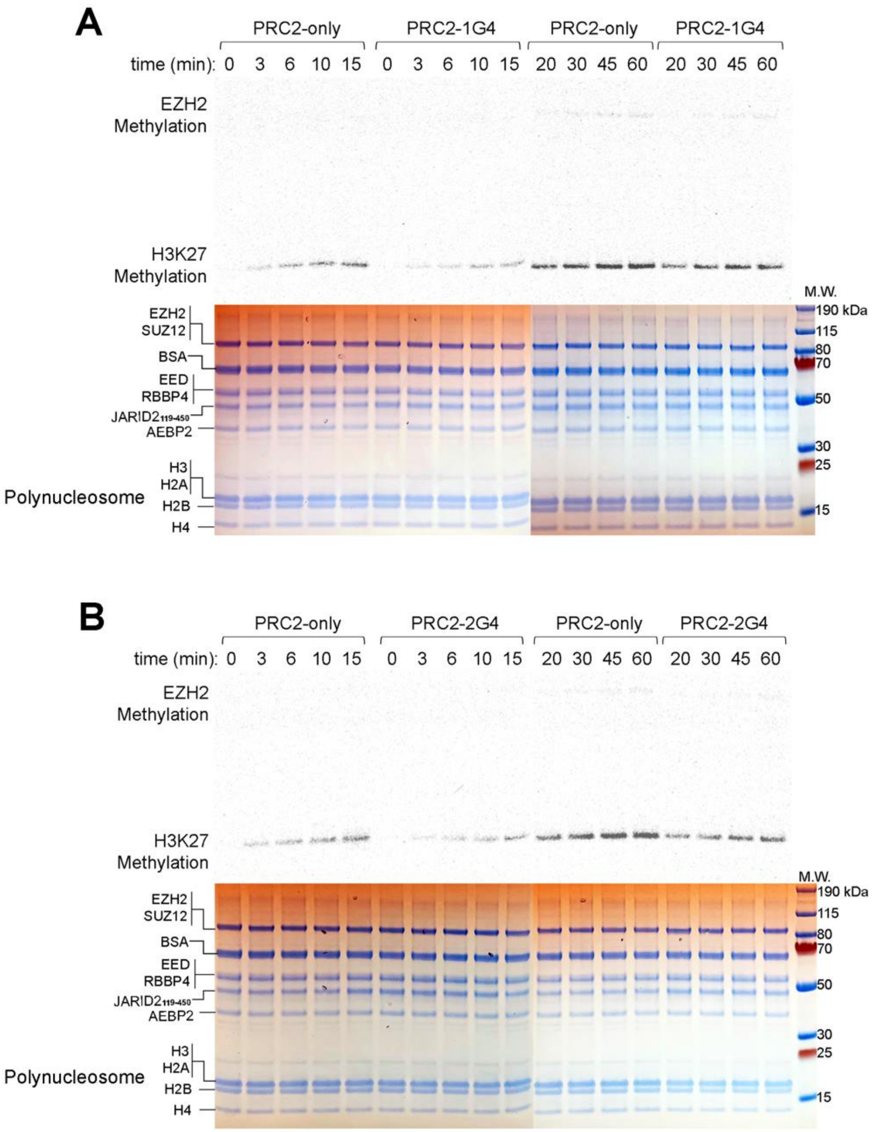

Fig. S12. Methyltransferase activity assay of six-subunit PRC2 with polynucleosomes as substrates. We incubated PRC2, polynucleosomes, radiolabeled S-adenosyl methionine (SAM), and corresponding RNA or mock for different reaction times. Signal intensities were quantified with replicates and plotted in **(**Fig. 3G**)**. Bottom images, Coomassie-staining of the same gels showing equal loading of each sample.

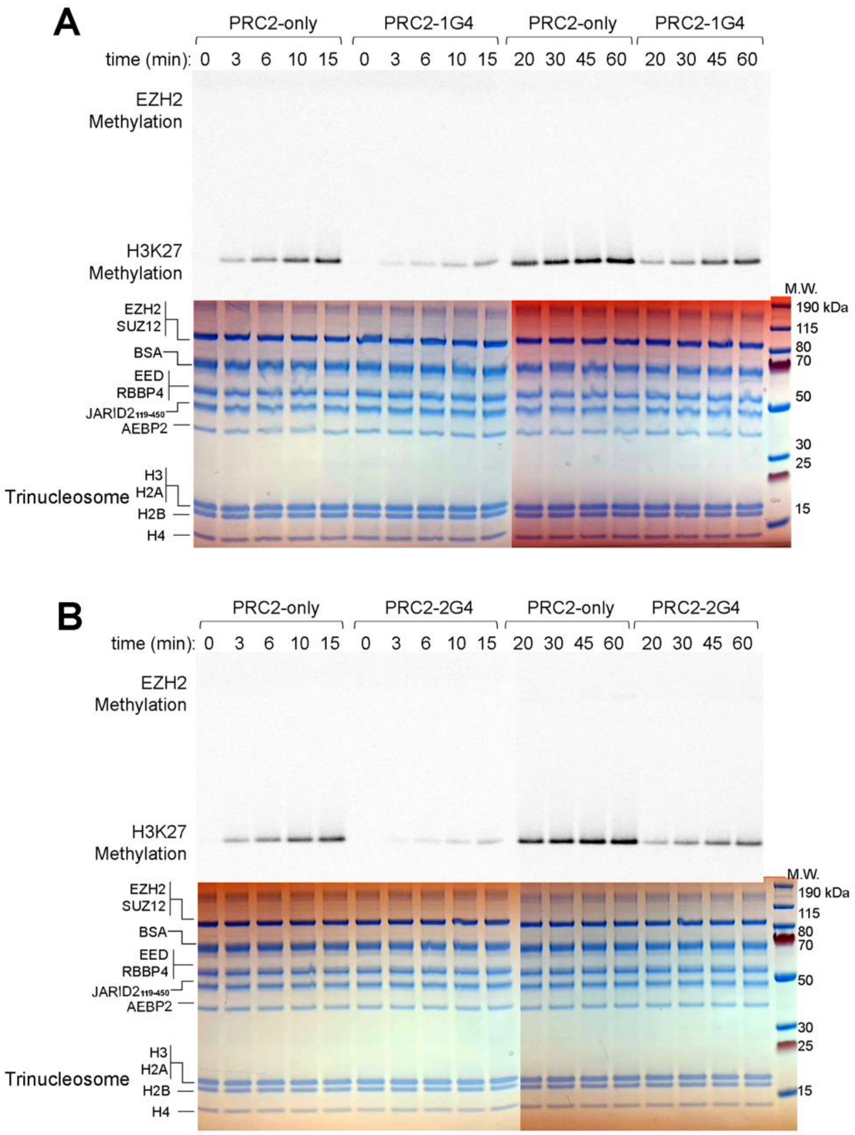

Fig. S13. Methyltransferase activity assay of six-subunit PRC2 with trinucleosomes as substrates. We incubated PRC2, trinucleosomes, radiolabeled SAM, and corresponding RNA or mock for different reaction times. Signal intensities were quantified with replicates and plotted in **(**Fig. 3G**)**. Bottom images, Coomassie-staining of the same gels showing equal loading of each sample.

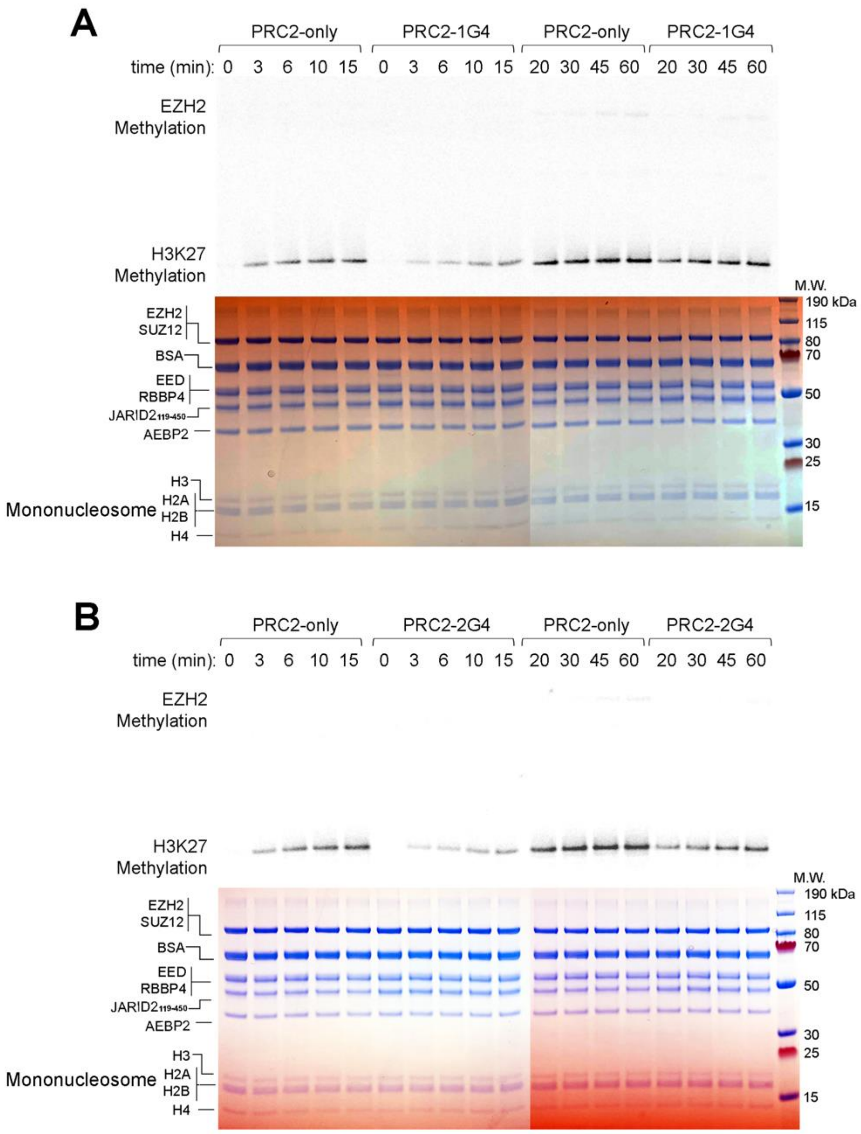

Fig. S14. Methyltransferase activity assay of six-subunit PRC2 with mononucleosomes as substrates. We incubated PRC2, mononucleosomes, radiolabeled SAM, and corresponding RNA or mock for different reaction times. Signal intensities were quantified with replicates and plotted in **(**Fig. 3G**)**. Bottom images, Coomassie-staining of the same gels showing equal loading of each sample.

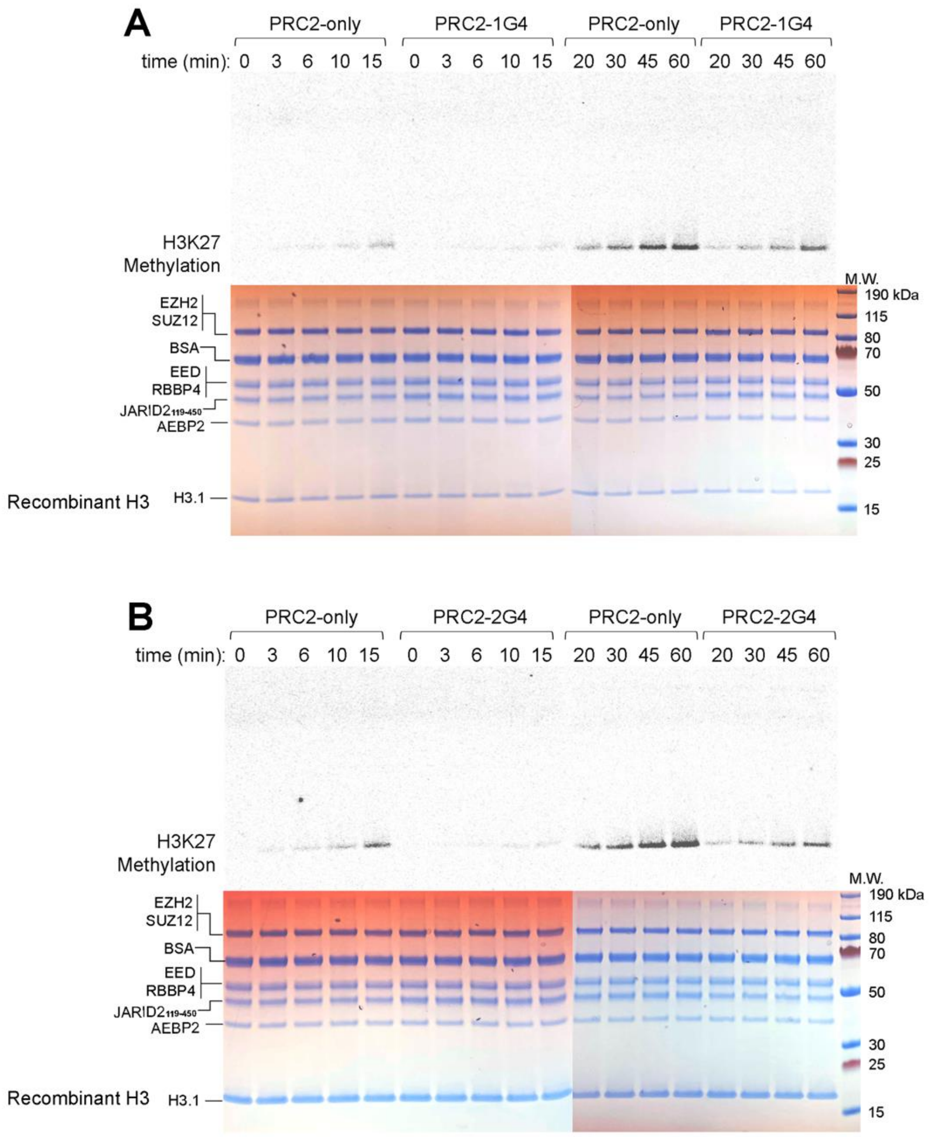

Fig. S15. Methyltransferase activity assay of six-subunit PRC2 with recombinant H3 as substrate. We incubated PRC2, recombinant H3.1, radiolabeled SAM, and corresponding RNA or mock for different reaction times. Signal intensities were quantified with replicates and plotted in **(**Fig. 3G**)**. Bottom images, Coomassie-staining of the same gels showing equal loading of each sample.

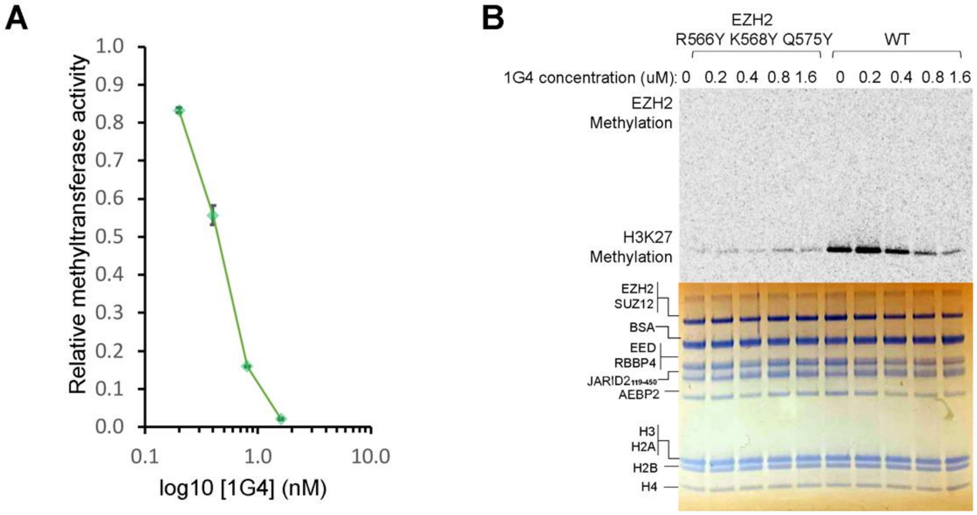

Fig. S16. Inhibition of histone methyltransferase activity by 1G4 RNA. (**A**) Methylation of trinucleosomes by PRC2 with serial dilutions of 1G4 RNA. Signal intensities were quantified and plotted against 1G4 concentration. Error bars are range of the values (n=2). (**B**) Methylation of trinucleosomes by EZH2 R566Y K568Y Q575Y and WT PRC2. We attempted to test the EZH2 R566Y K568Y Q575Y in response to RNA-mediated inhibition. However, this mutant had a basal level of activity that was too weak to be accurately quantified, presumably because those mutated residues are responsible for H3 tail loading of PRC2. This experiment was repeated three times with identical results.

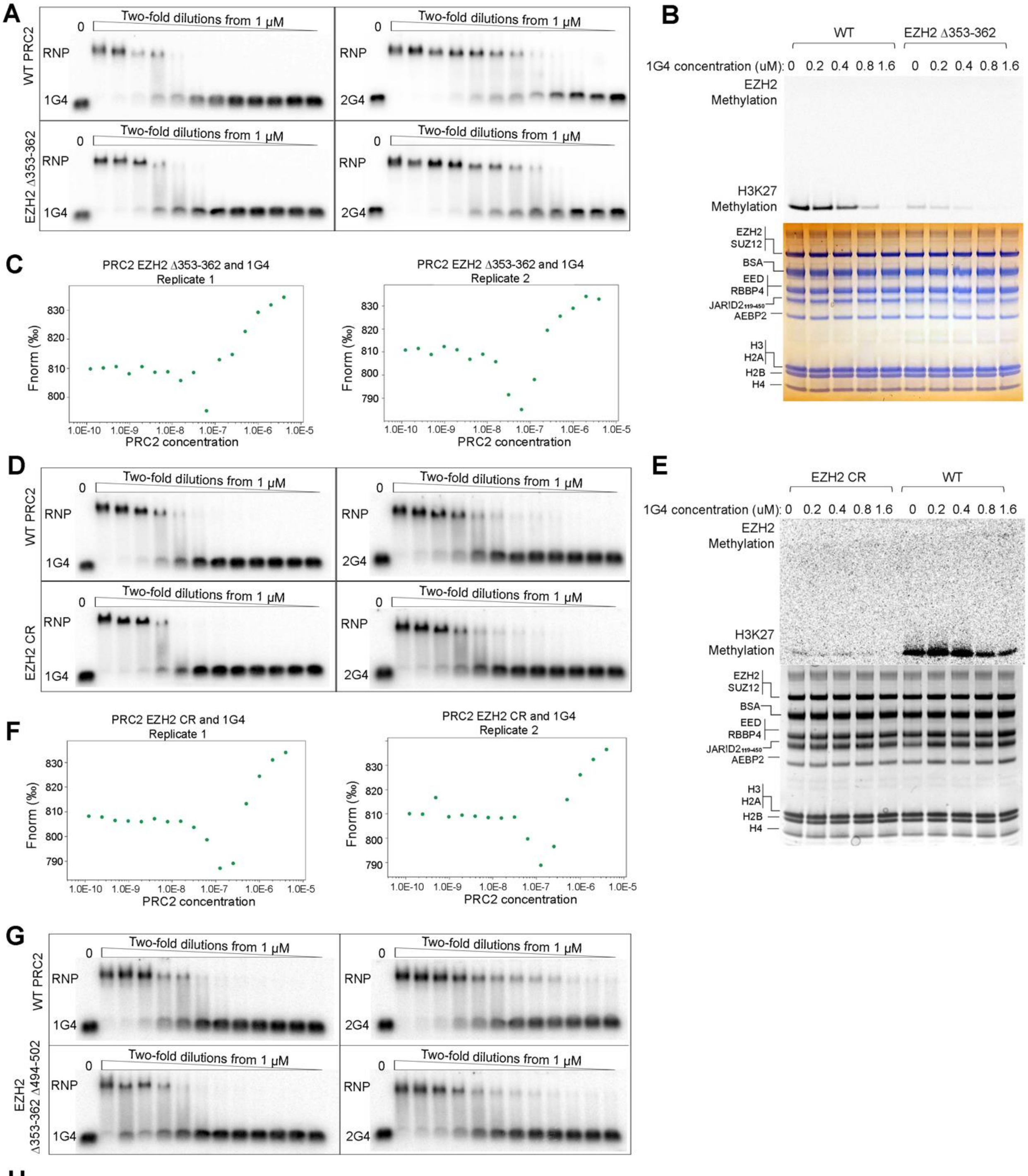

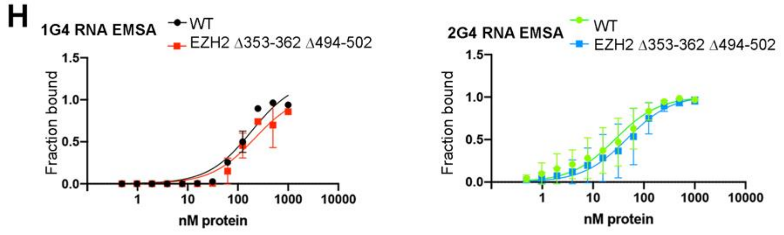

Kd	WT	EZH2 Δ353-362 Δ494-502
1G4	166 ± 67 nM	215 ± 84 nM
2G4	27 ± 8 nM	47 ± 15 nM

Fig. S17. RNA binding and HMTase activity of EZH2 Δ353-362, EZH2 CR and EZH2 Δ353-362 Δ494-502 mutants in vitro. (**A**) EMSA of WT and EZH2 Δ353-362 with 1G4 and 2G4 RNA. (**B**) Methyltransferase activity assay to compare WT PRC2 with EZH2 Δ353-362. Trinucleosome was the substrate. This experiment was repeated three times with identical results. (**C**) MST data showing a two-stage curve for EZH2 Δ353-362-1G4 interaction. (**D**) EMSA of WT and EZH2 CR mutant. (**E**) Methyltransferase activity assay to compare WT PRC2 with EZH2 CR. Trinucleosome was the substrate. This experiment was repeated four times with identical results. (**F**) MST data of EZH2 CR-1G4 interaction. (**G**) EMSA of WT and EZH2 Δ353-362 Δ494-502. (**H**) Quantification of three EMSA replicates. Error bars indicate SD of three replicates performed on different days.

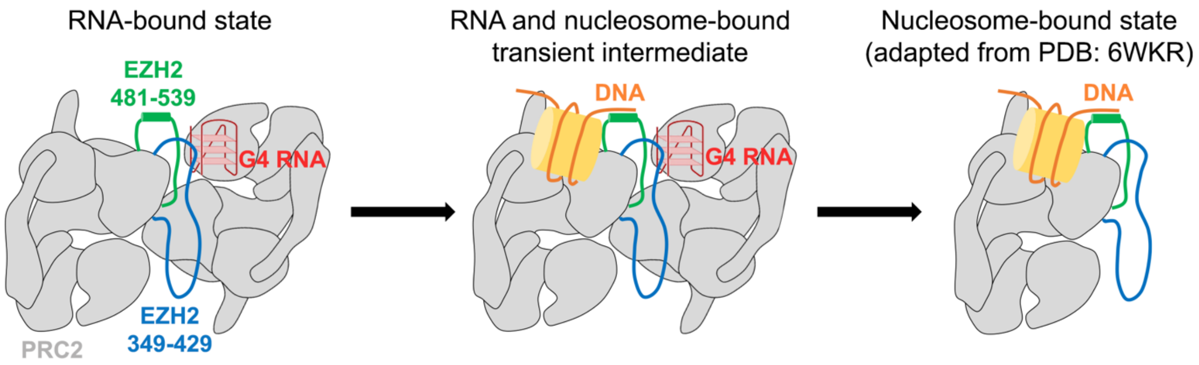

Fig. S18. PRC2 has the intrinsic ability to directly transfer from RNA to DNA without a free-enzyme intermediate, and the RNA-binding loop is involved in the transfer. Schematic representation of the direct transfer model and the proposed transient ternary complex of RNA and DNA bound to PRC2.

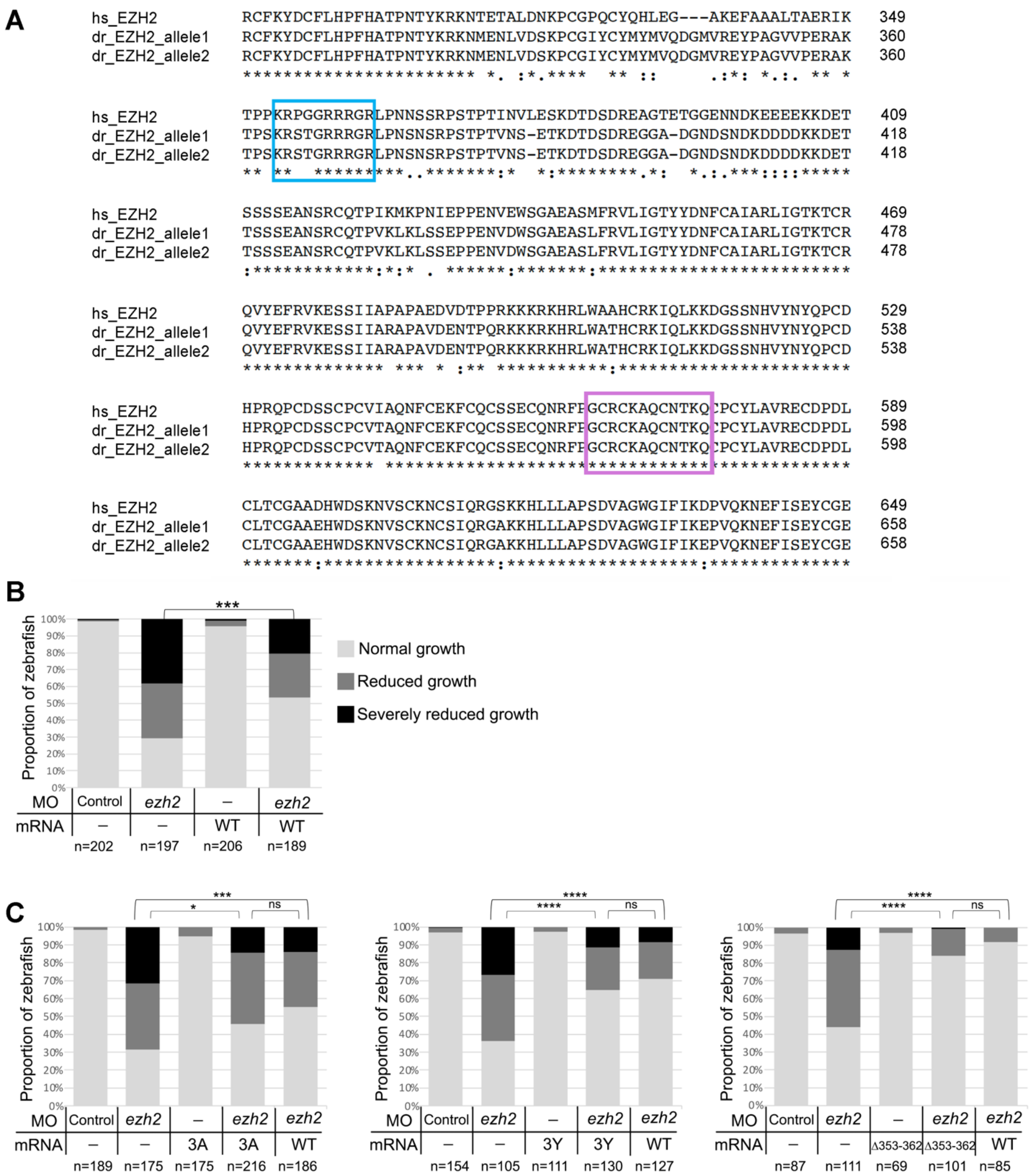

Fig. S19. Human EZH2 rescued the zebrafish growth defect. (**A**) Amino acid sequence alignment of human and zebrafish EZH2. EZH2 353-362 (RNA binding) and 564-575 (protein dimerization) sites are highlighted with blue and purple boxes correspondingly. *hs* is *Homo sapiens*; *dr* is *Danio rerio* (zebrafish). Zebrafish has two EZH2 alleles. (**B**) Scoring of anterior-posterior axis growth at 48 hpf. Zebrafish embryos were injected with 4 ng *ezh2*-MO or same amount of standard control MO. For rescue experiments, 100 ng mRNA encoding WT human EZH2 was co-injected. Fisher's exact test was used to determine the P values which are denoted

as ns; ∗, $p < 0.05$; ∗∗, $p < 0.01$; ∗∗∗, $p < 0.001$; ∗∗∗∗, $p < 0.0001$. (**C**) Scoring of anterior-posterior axis growth to examine functional complementation of EZH2 R566A K568A Q575A (3A), EZH2 R566Y K568Y Q575Y (3Y), and EZH2 Δ353-362 mutants. Fisher's exact test was used to determine the P values which are denoted as ns; ∗, $p < 0.05$; ∗∗, $p < 0.01$; ∗∗∗, $p < 0.001$; ∗∗∗∗, $p < 0.0001$.

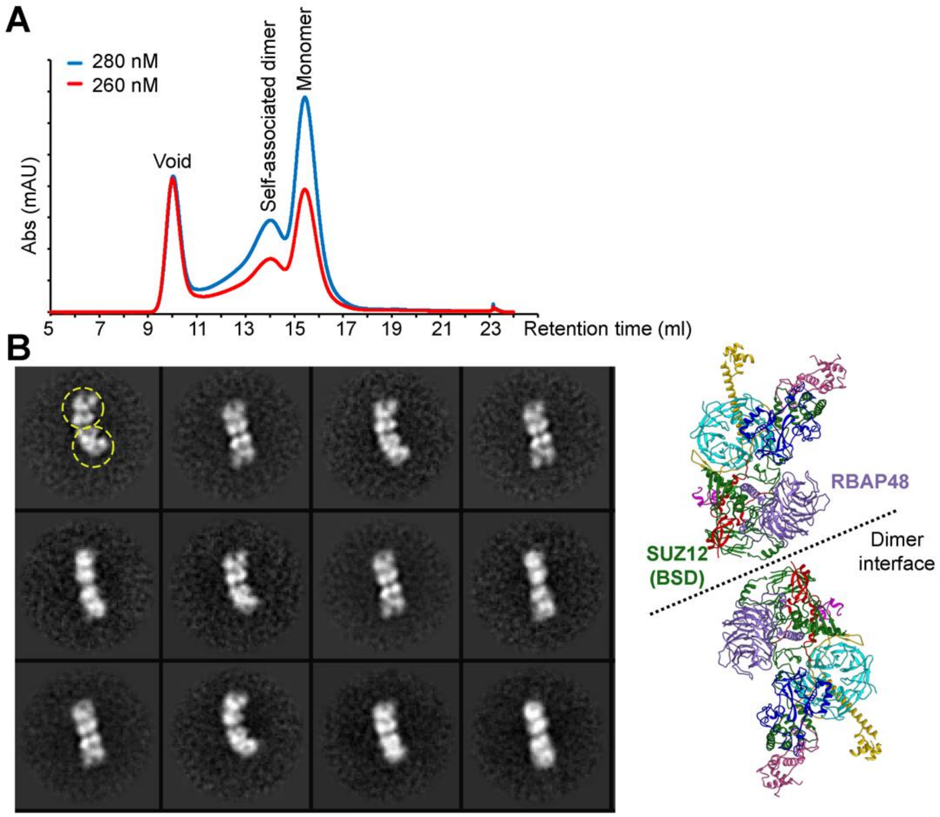

Fig. S20. PRC2 self-associated dimer has a different arrangement of protomers than the RNA-induced dimer. (**A**) We used size-exclusion purification on a Superose 6 increase 10/300 column as a polishing step in PRC2 purification, which consistently separated self-associated dimers. (**B**) Negative stain EM 2D class averages of self-associated PRC2 dimer exhibit a distinctive end-on dimer interface which is different from the RNA-induced dimer geometry. Yellow circles highlight two PRC2 protomers. The negative stain data was collected from a Tecnai F20 equipped with Gatan 4k x 4k CCD camera by Dr. Vignesh Kasinath in the laboratory of Dr. Eva Nogales at University of California, Berkeley. PRC2 proteins collected from the monomer peak of size-exclusion purification do not re-assemble to dimers (*26*).

Table S1. Cryo-EM data collection, refinement, and validation statistics

	Body1<br>EMD-29647	Body2<br>EMD-29656	Consensus<br>PDB: 8FYH<br>EMD-29578
**Data collection and Processing**			
Microscope	Krios	Krios	Krios
Voltage (keV)	300	300	300
Camera	K3	K3	K3
Magnification	81,000x	81,000x	81,000x
Pixel size at detector (Å/pixel)	0.844	0.844	0.844
Total electron exposure (e^{-}/Å^2)	60	60	60
Exposure rate (e^{-}/pixel/sec)	6.1	6.1	6.1
Number of frames collected during exposure	60	60	60
Defocus range (µm)	-0.6 to -2.0	-0.6 to -2.0	-0.6 to -2.0
Automation software (EPU, SerialEM or manual)	SerialEM	SerialEM	SerialEM
Energy filter slit width (eV)	20	20	20
Micrographs collected (no.)	18,632	18,632	18,632
Micrographs used (no.)	18,632	18,632	18,632
Total extracted particles (no.)	3,885,383	3,885,383	3,885,383
**For each reconstruction:**			
Refined particles (no.)	217,196	217,196	217,196
Final particles (no.)	217,196	217,196	217,196
Point-group or helical symmetry parameters	C1	C1	C1
Estimated error of translations/rotations	0.55/1.1	0.55/1.1	0.72/1.1
Resolution (global, Å)			
FSC 0.5 (unmasked/masked)	4.7/3.8	5.1/3.8	6.8/3.9
FSC 0.143 (unmasked/masked)	3.8/3.3	3.8/3.3	3.9/3.4
Resolution range (local, Å)	3.1-10.3	3.0-9.8	3.1-11.2
Map sharpening *B* factor (Å^2)	40	40	40
Map sharpening methods	RELION	RELION	RELION
**Model composition**			
Protein	6	6	12
Ligands	7	7	14
RNA/DNA	0	0	1
**Model Refinement**			
Refinement package	PHENIX	PHENIX	PHENIX
- real or reciprocal space	real space	real space	real space
- resolution cutoff (Å)	4	4	4
Model-Map scores			
-CC	0.8	0.8	0.72
- FSC $_{model\text{-}vs\text{-}map}$=0.5 (Å)	3.4	3.4	3.9
B factors (Å^2)	40	40	40
Protein residues	1846	1846	3692
Ligands	7	7	14
RNA/DNA (nucleotide)	0	0	18
R.m.s. deviations from ideal values			
Bond lengths (Å)	0.002	0.002	0.009
Bond angles (°)	0.495	0.459	0.628
**Validation**			
MolProbity score	1.74	1.73	1.99

CaBLAM outliers	4.97	4.97	4.97
Clashscore	5.74	5.21	10.75
Poor rotamers (%)	0.31	0.1	0.05
C-beta deviations	0	0	0.03
Ramachandran plot			
Favored (%)	93.38	92.99	93.16
Allowed (%)	6.4	6.73	6.62
Outliers (%)	0.22	0.28	0.22

Movie S1.

Cryo-EM structure of 1G4 RNA-bound PRC2 complex showing a close-up view of the PRC2 dimer interface and protein regions proximal to RNA.

Movie S2.

Structural comparison of the RNA-bound PRC2 dimer with the nucleosome-bound PRC2 to emphasize how dimerization inhibits PRC2 activity.

References and Notes

1. D. G Hendrickson, D. R. Kelley, D. Tenen, B. Bernstein, J. L. Rinn, Widespread RNA binding by chromatin-associated proteins. *Genome Biol.* **17**, 28 (2016). doi:10.1186/s13059-016-0878-3 Medline

2. Y. Long, X. Wang, D. T. Youmans, T. R. Cech, How do lncRNAs regulate transcription? *Sci. Adv.* **3**, eaao2110 (2017). doi:10.1126/sciadv.aao2110 Medline

3. R. Xiao, J.-Y. Chen, Z. Liang, D. Luo, G. Chen, Z. J. Lu, Y. Chen, B. Zhou, H. Li, X. Du, Y. Yang, M. San, X. Wei, W. Liu, E. Lécuyer, B. R. Graveley, G. W. Yeo, C. B. Burge, M. Q. Zhang, Y. Zhou, X.-D. Fu, Pervasive Chromatin-RNA Binding Protein Interactions Enable RNA-Based Regulation of Transcription. *Cell* **178**, 107–121.e18 (2019). doi:10.1016/j.cell.2019.06.001 Medline

4. C. Davidovich, T. R. Cech, The recruitment of chromatin modifiers by long noncoding RNAs: Lessons from PRC2. *RNA* **21**, 2007–2022 (2015). doi:10.1261/rna.053918.115 Medline

5. J. Yan, B. Dutta, Y. T. Hee, W. J. Chng, Towards understanding of PRC2 binding to RNA. *RNA Biol.* **16**, 176–184 (2019). doi:10.1080/15476286.2019.1565283 Medline

6. R. Cao, L. Wang, H. Wang, L. Xia, H. Erdjument-Bromage, P. Tempst, R. S. Jones, Y. Zhang, Role of histone H3 lysine 27 methylation in Polycomb-group silencing. *Science* **298**, 1039–1043 (2002). doi:10.1126/science.1076997 Medline

7. R. Margueron, D. Reinberg, The Polycomb complex PRC2 and its mark in life. *Nature* **469**, 343–349 (2011). doi:10.1038/nature09784 Medline

8. A. Laugesen, J. W. Højfeldt, K. Helin, Role of the Polycomb Repressive Complex 2 (PRC2) in Transcriptional Regulation and Cancer. *Cold Spring Harb. Perspect. Med.* **6**, a026575 (2016). doi:10.1101/cshperspect.a026575 Medline

9. S. Hauri, F. Comoglio, M. Seimiya, M. Gerstung, T. Glatter, K. Hansen, R. Aebersold, R. Paro, M. Gstaiger, C. Beisel, A High-Density Map for Navigating the Human Polycomb Complexome. *Cell Rep.* **17**, 583–595 (2016). doi:10.1016/j.celrep.2016.08.096 Medline

10. G. van Mierlo, G. J. C. Veenstra, M. Vermeulen, H. Marks, The Complexity of PRC2 Subcomplexes. *Trends Cell Biol.* **29**, 660–671 (2019). doi:10.1016/j.tcb.2019.05.004 Medline

11. J. Zhao, T. K. Ohsumi, J. T. Kung, Y. Ogawa, D. J. Grau, K. Sarma, J. J. Song, R. E. Kingston, M. Borowsky, J. T. Lee, Genome-wide identification of polycomb-associated RNAs by RIP-seq. *Mol. Cell* **40**, 939–953 (2010). doi:10.1016/j.molcel.2010.12.011 Medline

12. C. Davidovich, L. Zheng, K. J. Goodrich, T. R. Cech, Promiscuous RNA binding by Polycomb repressive complex 2. *Nat. Struct. Mol. Biol.* **20**, 1250–1257 (2013). doi:10.1038/nsmb.2679 Medline

13. M. Rosenberg, R. Blum, B. Kesner, E. Aeby, J.-M. Garant, A. Szanto, J. T. Lee, Motif-driven interactions between RNA and PRC2 are rheostats that regulate transcription elongation. *Nat. Struct. Mol. Biol.* **28**, 103–117 (2021). doi:10.1038/s41594-020-00535-9 Medline

14. S. Kaneko, J. Son, R. Bonasio, S. S. Shen, D. Reinberg, Nascent RNA interaction keeps PRC2 activity poised and in check. *Genes Dev.* **28**, 1983–1988 (2014). doi:10.1101/gad.247940.114 Medline

15. X. Wang, K. J. Goodrich, A. R. Gooding, H. Naeem, S. Archer, R. D. Paucek, D. T. Youmans, T. R. Cech, C. Davidovich, Targeting of Polycomb Repressive Complex 2 to RNA by Short Repeats of Consecutive Guanines. *Mol. Cell* **65**, 1056–1067.e5 (2017). doi:10.1016/j.molcel.2017.02.003 Medline

16. J. J. Montero, I. López-Silanes, D. Megías, M. F Fraga, Á. Castells-García, M. A. Blasco, TERRA recruitment of polycomb to telomeres is essential for histone trymethylation marks at telomeric heterochromatin. *Nat. Commun.* **9**, 1548 (2018). doi:10.1038/s41467-018-03916-3 Medline

17. D. Varshney, J. Spiegel, K. Zyner, D. Tannahill, S. Balasubramanian, The regulation and functions of DNA and RNA G-quadruplexes. *Nat. Rev. Mol. Cell Biol.* **21**, 459–474 (2020). doi:10.1038/s41580-020-0236-x Medline

18. C. Cifuentes-Rojas, A. J. Hernandez, K. Sarma, J. T. Lee, Regulatory interactions between RNA and polycomb repressive complex 2. *Mol. Cell* **55**, 171–185 (2014). doi:10.1016/j.molcel.2014.05.009 Medline

19. Y. Long, T. Hwang, A. R. Gooding, K. J. Goodrich, J. L. Rinn, T. R. Cech, RNA is essential for PRC2 chromatin occupancy and function in human pluripotent stem cells. *Nat. Genet.* **52**, 931–938 (2020). doi:10.1038/s41588-020-0662-x Medline

20. W. O. Hemphill, R. Fenske, A. R. Gooding, T. R. Cech, PRC2 direct transfer from G-quadruplex RNA to dsDNA has implications for RNA-binding chromatin modifiers. *Proc. Natl. Acad. Sci. U.S.A.* **120**, e2220528120 (2023). doi:10.1073/pnas.2220528120 Medline

21. S. Kaneko, J. Son, S. S. Shen, D. Reinberg, R. Bonasio, PRC2 binds active promoters and contacts nascent RNAs in embryonic stem cells. *Nat. Struct. Mol. Biol.* **20**, 1258–1264 (2013). doi:10.1038/nsmb.2700 Medline

22. M. Beltran, C. M. Yates, L. Skalska, M. Dawson, F. P. Reis, K. Viiri, C. L. Fisher, C. R. Sibley, B. M. Foster, T. Bartke, J. Ule, R. G. Jenner, The interaction of PRC2 with RNA or chromatin is mutually antagonistic. *Genome Res.* **26**, 896–907 (2016). doi:10.1101/gr.197632.115 Medline

23. M. Beltran, M. Tavares, N. Justin, G. Khandelwal, J. Ambrose, B. M. Foster, K. B. Worlock, A. Tvardovskiy, S. Kunzelmann, J. Herrero, T. Bartke, S. J. Gamblin, J. R. Wilson, R. G. Jenner, G-tract RNA removes Polycomb repressive complex 2 from genes. *Nat. Struct. Mol. Biol.* **26**, 899–909 (2019). doi:10.1038/s41594-019-0293-z Medline

24. X. Wang, R. D. Paucek, A. R. Gooding, Z. Z. Brown, E. J. Ge, T. W. Muir, T. R. Cech, Molecular analysis of PRC2 recruitment to DNA in chromatin and its inhibition by RNA. *Nat. Struct. Mol. Biol.* **24**, 1028–1038 (2017). doi:10.1038/nsmb.3487 Medline

25. Q. Zhang, N. J. McKenzie, R. Warneford-Thomson, E. H. Gail, S. F. Flanigan, B. M. Owen, R. Lauman, V. Levina, B. A. Garcia, R. B. Schittenhelm, R. Bonasio, C. Davidovich, RNA exploits an exposed regulatory site to inhibit the enzymatic activity of PRC2. *Nat. Struct. Mol. Biol.* **26**, 237–247 (2019). doi:10.1038/s41594-019-0197-y Medline

26. V. Kasinath, M. Faini, S. Poepsel, D. Reif, X. A. Feng, G. Stjepanovic, R. Aebersold, E. Nogales, Structures of human PRC2 with its cofactors AEBP2 and JARID2. *Science* **359**, 940–944 (2018). doi:10.1126/science.aar5700 Medline

27. V. Kasinath, C. Beck, P. Sauer, S. Poepsel, J. Kosmatka, M. Faini, D. Toso, R. Aebersold, E. Nogales, JARID2 and AEBP2 regulate PRC2 in the presence of H2AK119ub1 and other histone modifications. *Science* **371**, eabc3393 (2021). doi:10.1126/science.abc3393 Medline

28. S. Poepsel, V. Kasinath, E. Nogales, Cryo-EM structures of PRC2 simultaneously engaged with two functionally distinct nucleosomes. *Nat. Struct. Mol. Biol.* **25**, 154–162 (2018). doi:10.1038/s41594-018-0023-y Medline

29. D. Grau, Y. Zhang, C.-H. Lee, M. Valencia-Sánchez, J. Zhang, M. Wang, M. Holder, V. Svetlov, D. Tan, E. Nudler, D. Reinberg, T. Walz, K.-J. Armache, Structures of monomeric and dimeric PRC2:EZH1 reveal flexible modules involved in chromatin compaction. *Nat. Commun.* **12**, 714 (2021). doi:10.1038/s41467-020-20775-z Medline

30. L. Gong, X. Liu, L. Jiao, X. Yang, A. Lemoff, X. Liu, CK2-mediated phosphorylation of SUZ12 promotes PRC2 function by stabilizing enzyme active site. *Nat. Commun.* **13**, 6781 (2022). doi:10.1038/s41467-022-34431-1 Medline

31. L. Jiao, X. Liu, Structural basis of histone H3K27 trimethylation by an active polycomb repressive complex 2. *Science* **350**, aac4383 (2015). doi:10.1126/science.aac4383 Medline

32. S. Chen, L. Jiao, X. Liu, X. Yang, X. Liu, A Dimeric Structural Scaffold for PRC2-PCL Targeting to CpG Island Chromatin. *Mol. Cell* **77**, 1265–1278.e7 (2020). doi:10.1016/j.molcel.2019.12.019 Medline

33. S. Chen, L. Jiao, M. Shubbar, X. Yang, X. Liu, Unique Structural Platforms of Suz12 Dictate Distinct Classes of PRC2 for Chromatin Binding. *Mol. Cell* **69**, 840–852.e5 (2018). doi:10.1016/j.molcel.2018.01.039 Medline

34. K. Finogenova, J. Bonnet, S. Poepsel, I. B. Schäfer, K. Finkl, K. Schmid, C. Litz, M. Strauss, C. Benda, J. Müller, Structural basis for PRC2 decoding of active histone methylation marks H3K36me2/3. *eLife* **9**, e61964 (2020). doi:10.7554/eLife.61964 Medline

35. B. G. Han, Z. Watson, H. Kang, A. Pulk, K. H. Downing, J. Cate, R. M. Glaeser, Long shelf-life streptavidin support-films suitable for electron microscopy of biological macromolecules. *J. Struct. Biol.* **195**, 238–244 (2016). doi:10.1016/j.jsb.2016.06.009 Medline

36. T. Nakane, S. H. W. Scheres, Multi-body Refinement of Cryo-EM Images in RELION. *Methods Mol. Biol.* **2215**, 145–160 (2021). doi:10.1007/978-1-0716-0966-8_7 Medline

37. V. Kasinath, S. Poepsel, E. Nogales, Recent Structural Insights into Polycomb Repressive Complex 2 Regulation and Substrate Binding. *Biochemistry* **58**, 346–354 (2019). doi:10.1021/acs.biochem.8b01064 Medline

38. X. C. Bai, E. Rajendra, G. Yang, Y. Shi, S. H. Scheres, Sampling the conformational space of the catalytic subunit of human γ-secretase. *eLife* **4**, e11182 (2015). doi:10.7554/eLife.11182 Medline

39. S. Kaneko, G. Li, J. Son, C.-F. Xu, R. Margueron, T. A. Neubert, D. Reinberg, Phosphorylation of the PRC2 component Ezh2 is cell cycle-regulated and up-regulates its binding to ncRNA. *Genes Dev.* **24**, 2615–2620 (2010). doi:10.1101/gad.1983810 Medline

40. Y. Long, B. Bolanos, L. Gong, W. Liu, K. J. Goodrich, X. Yang, S. Chen, A. R. Gooding, K. A. Maegley, K. S. Gajiwala, A. Brooun, T. R. Cech, X. Liu, Conserved RNA-binding specificity of polycomb repressive complex 2 is achieved by dispersed amino acid patches in EZH2. *eLife* **6**, e31558 (2017). doi:10.7554/eLife.31558 Medline

41. M. A. Lemmon, Z. Bu, J. E. Ladbury, M. Zhou, D. Pinchasi, I. Lax, D. M. Engelman, J. Schlessinger, Two EGF molecules contribute additively to stabilization of the EGFR dimer. *EMBO J.* **16**, 281–294 (1997). doi:10.1093/emboj/16.2.281 Medline

42. O. Oksuz, J. E. Henninger, R. Warneford-Thomson, M. M. Zheng, H. Erb, A. Vancura, K. J. Overholt, S. W. Hawken, S. F. Banani, R. Lauman, L. N. Reich, A. L. Robertson, N. M. Hannett, T. I. Lee, L. I. Zon, R. Bonasio, R. A. Young, Transcription factors interact with RNA to regulate genes. *Mol. Cell* **83**, 2449–2463.e13 (2023). doi:10.1016/j.molcel.2023.06.012

43. S. Kaneko, R. Bonasio, R. Saldaña-Meyer, T. Yoshida, J. Son, K. Nishino, A. Umezawa, D. Reinberg, Interactions between JARID2 and noncoding RNAs regulate PRC2 recruitment to chromatin. *Mol. Cell* **53**, 290–300 (2014). doi:10.1016/j.molcel.2013.11.012 Medline

44. W. O. Hemphill, C. K. Voong, R. Fenske, J. A. Goodrich, T. R. Cech, Multiple RNA- and DNA-binding proteins exhibit direct transfer of polynucleotides with implications for target-site search. *Proc. Natl. Acad. Sci. U.S.A.* **120**, e2220537120 (2023). doi:10.1073/pnas.2220537120 Medline

45. Y. Zhong, Q. Ye, C. Chen, M. Wang, H. Wang, Ezh2 promotes clock function and hematopoiesis independent of histone methyltransferase activity in zebrafish. *Nucleic Acids Res.* **46**, 3382–3399 (2018). doi:10.1093/nar/gky101 Medline

46. C. J. Sneeringer, M. P. Scott, K. W. Kuntz, S. K. Knutson, R. M. Pollock, V. M. Richon, R. A. Copeland, Coordinated activities of wild-type plus mutant EZH2 drive tumor-associated hypertrimethylation of lysine 27 on histone H3 (H3K27) in human B-cell lymphomas. *Proc. Natl. Acad. Sci. U.S.A.* **107**, 20980–20985 (2010). doi:10.1073/pnas.1012525107 Medline

47. D. B. Yap, J. Chu, T. Berg, M. Schapira, S.-W. G. Cheng, A. Moradian, R. D. Morin, A. J. Mungall, B. Meissner, M. Boyle, V. E. Marquez, M. A. Marra, R. D. Gascoyne, R. K. Humphries, C. H. Arrowsmith, G. B. Morin, S. A. J. R. Aparicio, Somatic mutations at EZH2 Y641 act dominantly through a mechanism of selectively altered PRC2 catalytic activity, to increase H3K27 trimethylation. *Blood* **117**, 2451–2459 (2011). doi:10.1182/blood-2010-11-321208 Medline

48. G. P. Souroullas, W. R. Jeck, J. S. Parker, J. M. Simon, J.-Y. Liu, J. Paulk, J. Xiong, K. S. Clark, Y. Fedoriw, J. Qi, C. E. Burd, J. E. Bradner, N. E. Sharpless, An oncogenic Ezh2 mutation induces tumors through global redistribution of histone 3 lysine 27 trimethylation. *Nat. Med.* **22**, 632–640 (2016). doi:10.1038/nm.4092 Medline

49. S. Sanulli, N. Justin, A. Teissandier, K. Ancelin, M. Portoso, M. Caron, A. Michaud, B. Lombard, S. T. da Rocha, J. Offer, D. Loew, N. Servant, M. Wassef, F. Burlina, S. J. Gamblin, E. Heard, R. Margueron, Jarid2 Methylation via the PRC2 Complex Regulates H3K27me3 Deposition during Cell Differentiation. *Mol. Cell* **57**, 769–783 (2015). doi:10.1016/j.molcel.2014.12.020 Medline

50. Q. Zhang, S. C. Agius, S. F. Flanigan, M. Uckelmann, V. Levina, B. M. Owen, C. Davidovich, PALI1 facilitates DNA and nucleosome binding by PRC2 and triggers an allosteric activation of catalysis. *Nat. Commun.* **12**, 4592 (2021). doi:10.1038/s41467-021-24866-3 Medline

51. A. Laugesen, J. W. Højfeldt, K. Helin, Molecular Mechanisms Directing PRC2 Recruitment and H3K27 Methylation. *Mol. Cell* **74**, 8–18 (2019). doi:10.1016/j.molcel.2019.03.011 Medline

52. C. Davidovich, K. J. Goodrich, A. R. Gooding, T. R. Cech, A dimeric state for PRC2. *Nucleic Acids Res.* **42**, 9236–9248 (2014). doi:10.1093/nar/gku540 Medline

53. R. Saldaña-Meyer, J. Rodriguez-Hernaez, T. Escobar, M. Nishana, K. Jácome-López, E. P. Nora, B. G. Bruneau, A. Tsirigos, M. Furlan-Magaril, J. Skok, D. Reinberg, RNA Interactions Are Essential for CTCF-Mediated Genome Organization. *Mol. Cell* **76**, 412–422.e5 (2019). doi:10.1016/j.molcel.2019.08.015 Medline

54. H. R. Steiner, N. C. Lammer, R. T. Batey, D. S. Wuttke, An Extended DNA Binding Domain of the Estrogen Receptor Alpha Directly Interacts with RNAs *in Vitro*. *Biochemistry* **61**, 2490–2494 (2022). doi:10.1021/acs.biochem.2c00536 Medline

55. A. Di Ruscio, A. K. Ebralidze, T. Benoukraf, G. Amabile, L. A. Goff, J. Terragni, M. E. Figueroa, L. L. De Figueiredo Pontes, M. Alberich-Jorda, P. Zhang, M. Wu, F. D'Alò, A. Melnick, G. Leone, K. K. Ebralidze, S. Pradhan, J. L. Rinn, D. G. Tenen, DNMT1-interacting RNAs block gene-specific DNA methylation. *Nature* **503**, 371–376 (2013). doi:10.1038/nature12598 Medline

56. L. I. Jansson-Fritzberg, C. I. Sousa, M. J. Smallegan, J. J. Song, A. R. Gooding, V. Kasinath, J. L. Rinn, T. R. Cech, DNMT1 inhibition by pUG-fold quadruplex RNA. *RNA* 29, 346–360 (2023). Medline

57. S. Q. Zheng, E. Palovcak, J.-P. Armache, K. A. Verba, Y. Cheng, D. A. Agard, MotionCor2: Anisotropic correction of beam-induced motion for improved cryo-electron microscopy. *Nat. Methods* **14**, 331–332 (2017). doi:10.1038/nmeth.4193 Medline

58. D. Kimanius, L. Dong, G. Sharov, T. Nakane, S. H. W. Scheres, New tools for automated cryo-EM single-particle analysis in RELION-4.0. *Biochem. J.* **478**, 4169–4185 (2021). doi:10.1042/BCJ20210708 Medline

59. A. Rohou, N. Grigorieff, CTFFIND4: Fast and accurate defocus estimation from electron micrographs. *J. Struct. Biol.* **192**, 216–221 (2015). doi:10.1016/j.jsb.2015.08.008 Medline

60. D. Tegunov, L. Xue, C. Dienemann, P. Cramer, J. Mahamid, Multi-particle cryo-EM refinement with M visualizes ribosome-antibiotic complex at 3.5 Å in cells. *Nat. Methods* **18**, 186–193 (2021). doi:10.1038/s41592-020-01054-7 Medline

61. P. Emsley, K. Cowtan, Coot: Model-building tools for molecular graphics. *Acta Crystallogr. D Biol. Crystallogr.* **60**, 2126–2132 (2004). doi:10.1107/S0907444904019158 Medline

62. P. D. Adams, R. W. Grosse-Kunstleve, L.-W. Hung, T. R. Ioerger, A. J. McCoy, N. W. Moriarty, R. J. Read, J. C. Sacchettini, N. K. Sauter, T. C. Terwilliger, PHENIX: Building new software for automated crystallographic structure determination. *Acta Crystallogr. D Biol. Crystallogr.* **58**, 1948–1954 (2002). doi:10.1107/S0907444902016657 Medline

63. V. B. Chen, W. B. Arendall 3rd, J. J. Headd, D. A. Keedy, R. M. Immormino, G. J. Kapral, L. W. Murray, J. S. Richardson, D. C. Richardson, MolProbity: All-atom structure validation for macromolecular crystallography. *Acta Crystallogr. D Biol. Crystallogr.* **66**, 12–21 (2010). doi:10.1107/S0907444909042073 Medline

64. E. F. Pettersen, T. D. Goddard, C. C. Huang, G. S. Couch, D. M. Greenblatt, E. C. Meng, T. E. Ferrin, UCSF Chimera—A visualization system for exploratory research and analysis. *J. Comput. Chem.* **25**, 1605–1612 (2004). doi:10.1002/jcc.20084 Medline

65. J. Jumper, R. Evans, A. Pritzel, T. Green, M. Figurnov, O. Ronneberger, K. Tunyasuvunakool, R. Bates, A. Žídek, A. Potapenko, A. Bridgland, C. Meyer, S. A. A. Kohl, A. J. Ballard, A. Cowie, B. Romera-Paredes, S. Nikolov, R. Jain, J. Adler, T. Back, S. Petersen, D. Reiman, E. Clancy, M. Zielinski, M. Steinegger, M. Pacholska, T. Berghammer, S. Bodenstein, D. Silver, O. Vinyals, A. W. Senior, K. Kavukcuoglu, P. Kohli, D. Hassabis, Highly accurate protein structure prediction with AlphaFold. *Nature* **596**, 583–589 (2021). doi:10.1038/s41586-021-03819-2 Medline

66. W. Shao, R. Sharma, M. H. Clausen, H. V. Scheller, Microscale thermophoresis as a powerful tool for screening glycosyltransferases involved in cell wall biosynthesis. *Plant Methods* **16**, 99 (2020). doi:10.1186/s13007-020-00641-1 Medline

67. M. Asmari, R. Ratih, H. A. Alhazmi, S. El Deeb, Thermophoresis for characterizing biomolecular interaction. *Methods* **146**, 107–119 (2018). doi:10.1016/j.ymeth.2018.02.003 Medline

68. J. R. Williamson, M. K. Raghuraman, T. R. Cech, Monovalent cation-induced structure of telomeric DNA: The G-quartet model. *Cell* **59**, 871–880 (1989). doi:10.1016/0092-8674(89)90610-7 Medline
