# Supplementary material for: Structural basis for inactivation of PRC2 by G-quadruplex RNA
Source: Science. Author manuscript; Available in PMC 2024 Jun 21. (PMC11191771; doi:10.1126/science.adh0059)
Supplement: Reproducibility Checklist [file NIHMS1998120-supplement-Reproducibility_Checklist.docx]

**Materials Design Analysis Reporting (MDAR)**

**Checklist for Authors**

The MDAR framework establishes a minimum set of requirements in transparent reporting applicable to studies in the life sciences (see Statement of Task: doi:10.31222/osf.io/9sm4x.). The MDAR checklist is a tool for authors, editors, and others seeking to adopt the MDAR framework for transparent reporting in manuscripts and other outputs. Please refer to the MDAR Elaboration Document for additional context for the MDAR framework.

**For all that apply, please note where in the manuscript the required information is provided.**

**Materials:**

| **Newly created materials** | **indicate where provided: page no/section/legend)** | **n/a** |
| --- | --- | --- |
| The manuscript includes a dedicated "materials availability statement" providing transparent disclosure about availability of newly created materials including details on how materials can be accessed and describing any restrictions on access. | Page 11, Data and materials availability. |  |
|  |  |  |
| **Antibodies** | **indicate where provided: page no/section/legend)** | **n/a** |
| For commercial reagents, provide supplier name, catalogue number and [RRID](https://scicrunch.org/resources), if available. |  | √ |
|  |  |  |
| **DNA and RNA sequences** | **indicate where provided: page no/section/legend)** | **n/a** |
| **Short novel DNA or RNA including primers, probes:** Sequences should be included or deposited in a public repository. | Fig 1B includes sequences of two RNA oligos;  Supplementary materials, page 8, Materials and Methods, Zebrafish morpholino and mRNA rescue injections section includes sequences of two DNA morpholino oligos. |  |
|  |  |  |
| **Cell materials** | **indicate where provided: page no/section/legend** | **n/a** |
| **Cell lines:** Provide species information, strain. Provide accession number in repository **OR** supplier name, catalog number, clone number, **OR** RRID. | Supplementary materials, page 2, Materials and Methods, Portein expression and purification section includes two insect cell lines Sf9 (*Spodoptera frugiperda*, IPLB-Sf-21-AE) and HighFive (*Trichoplusia ni*, BTI-Tn-5B1-4). |  |
| **Primary cultures:** Provide species, strain, sex of origin, genetic modification status. |  | √ |
|  |  |  |
| **Experimental animals** | **indicate where provided: page no/section/legend)** | **n/a** |
| **Laboratory animals or Model organisms:** Provide species, strain, sex, age, genetic modification status. Provide accession number in repository **OR** supplier name, catalog number, clone number, **OR** RRID. | Supplementary materials, page 8, Materials and Methods, Zebrafish morpholino and mRNA rescue injections. |  |
| **Animal observed in or captured from the field:** Provide species, sex, and age where possible. |  | √ |
|  |  |  |
| **Plants and microbes** | **indicate where provided: page no/section/legend)** | **n/a** |
| **Plants:** provide species and strain, ecotype and cultivar where relevant, unique accession number if available, and source (including location for collected wild specimens). |  | √ |
| **Microbes:** provide species and strain, unique accession number if available, and source. |  | √ |
|  |  |  |
| **Human research participants** | **indicate where provided: page no/section/legend) or state if these demographics were not collected** | **n/a** |
| If collected and within the bounds of privacy constraints report on age, sex and gender or ethnicity for all study participants. |  | √ |

**Design:**

| **Study protocol** | **indicate where provided: page no/section/legend)** | **n/a** |
| --- | --- | --- |
| If study protocol has been pre-registered, provide DOI. For clinical trials, provide the trial registration number **OR** cite DOI. |  | √ |
|  |  |  |
| **Laboratory protocol** | **indicate where provided: page no/section/legend)** | **n/a** |
| Provide DOI **OR** other citation details if detailed step-by-step protocols are available. | Supplementary materials, Materials and Methods. |  |
|  |  |  |
| **Experimental study design (statistics details)** | | |
| **For in vivo studies:** State whether and how the following have been done | **indicate where provided: page no/section/legend. If it could have been done, but was not, write not done** | **n/a** |
| Sample size determination | Fig 4E and Fig 4F; Fig 4E legend; Supplementary materials, page 8, Materials and Methods, Zebrafish morpholino and mRNA rescue injections. |  |
| Randomisation | Supplementary materials, page 8, Materials and Methods, Zebrafish morpholino and mRNA rescue injections. |  |
| Blinding | Supplementary materials, page 8, Materials and Methods, Zebrafish morpholino and mRNA rescue injections. |  |
| Inclusion/exclusion criteria | Fig 4C and Fig 4C legend; Supplementary materials, page 8, Materials and Methods, Zebrafish morpholino and mRNA rescue injections. |  |
|  |  |  |
| **Sample definition and in-laboratory replication** | **indicate where provided: page no/section/legend** | **n/a** |
| State number of times the experiment was replicated in laboratory. | Fig 3G legend; Supplementary materials, Materials and Methods, Zebrafish morpholino and mRNA rescue injections; Supplementary materials, Fig S1B legend; Fig S8 legend; Fig S10B legend; Fig S10C legend; Fig S11B legend; Fig S16 legend; Fig S17B legend; Fig S17E legend; Fig S17H legend. |  |
| Define whether data describe technical or biological replicates. | Supplementary materials, Materials and Methods, Zebrafish morpholino and mRNA rescue injections section defines in vivo experiments as biological replicates; Other sections of Materials and Methods describe in vitro experiments as technical replicates. |  |
|  |  |  |
| **Ethics** | **indicate where provided: page no/section/legend** | **n/a** |
| **Studies involving human participants:** State details of authority granting ethics approval (IRB or equivalent committee(s), provide reference number for approval. |  | √ |
| **Studies involving experimental animals:** State details of authority granting ethics approval (IRB or equivalent committee(s), provide reference number for approval. | Supplementary materials, page 8, Materials and Methods, Zebrafish morpholino and mRNA rescue injections. |  |
| **Studies involving specimen and field samples:** State if relevant permits obtained, provide details of authority approving study; if none were required, explain why. |  | √ |
|  |  |  |
| **Dual Use Research of Concern (DURC)** | **indicate where provided: page no/section/legend** | **n/a** |
| If study is subject to dual use research of concern regulations, state the authority granting approval and reference number for the regulatory approval. |  | √ |

**Analysis:**

| **Attrition** | **indicate where provided: page no/section/legend** | **n/a** |
| --- | --- | --- |
| Describe whether exclusion criteria were preestablished. Report if sample or data points were omitted from analysis. If yes report if this was due to attrition or intentional exclusion and provide justification. |  | √ |
|  |  |  |
| **Statistics** | **indicate where provided: page no/section/legend** | **n/a** |
| Describe statistical tests used and justify choice of tests. | Fig 3G legend; Fig 4E legend; Supplementary materials, Materials and methods, Electrophoretic mobility shift assay (EMSA); Materials and methods, Fluorescence polarization (FP); Materials and Methods, Zebrafish morpholino and mRNA rescue injections; Supplementary materials, Fig S1B legend; Fig S10B legend; Fig S11B legend; Fig S16A legend; Fig S17H legend. Fig S19B legend; Fig S19C legend. |  |
|  |  |  |
| **Data availability** | **indicate where provided: page no/section/legend** | **n/a** |
| For newly created and reused datasets, the manuscript includes a data availability statement that provides details for access or notes restrictions on access. | Page 11, Data and materials availability. |  |
| If newly created datasets are publicly available, provide accession number in repository **OR** DOI **OR** URL and licensing details where available. | Page 11, Data and materials availability.  ‘Cryo-EM density maps and fitted models have been deposited in the Electron Microscopy Data Bank (EMD-29578, consensus map) (EMD-29647, Body1 from multibody refinement) (EMD-29656, Body2 from multibody refinement) and the Protein Data Bank (PDB: 8FYH).’ |  |
| If reused data is publicly available provide accession number in repository **OR** DOI **OR** URL, **OR** citation. |  | √ |
|  |  |  |
| **Code availability** | **indicate where provided: page no/section/legend** | **n/a** |
| For all newly generated custom computer code/software/mathematical algorithm or re-used code essential for replicating the main findings of the study, the manuscript includes a data availability statement that provides details for access or notes restrictions. |  | √ |
| If newly generated code is publicly available, provide accession number in repository, **OR** DOI **OR** URL and licensing details where available. State any restrictions on code availability or accessibility. |  | √ |
| If reused code is publicly available provide accession number in repository **OR** DOI **OR** URL, **OR** citation. |  | √ |

**Reporting**

MDAR framework recommends adoption of discipline-specific guidelines, established and endorsed through community initiatives. Journals have their own policy about requiring specific guidelines and recommendations to complement MDAR.

| **Adherence to community standards** | **indicate where provided: page no/section/legend** | **n/a** |
| --- | --- | --- |
| State if relevant guidelines (e.g., ICMJE, MIBBI, ARRIVE) have been followed, and whether a checklist (e.g., CONSORT, PRISMA, ARRIVE) is provided with the manuscript. |  | √ |
